# A randomized crossover trial comparing the Nifty cup to a medicine cup in preterm infants who have difficulty breastfeeding at Komfo Anokye Teaching Hospital (KATH) in Kumasi, Ghana

**DOI:** 10.1371/journal.pone.0223951

**Published:** 2019-10-17

**Authors:** Christy M. McKinney, Gyikua Plange-Rhule, Daniel Ansong, Michael L. Cunningham, Irene Agyeman, Patricia S. Coffey

**Affiliations:** 1 Division of Craniofacial Medicine, Department of Pediatrics, School of Medicine, University of Washington, Seattle Children’s Research Institute, and Seattle Children’s Hospital Craniofacial Center, Seattle, Washington, United States of America; 2 Komfo Anokye Teaching Hospital, Kwame Nkrumah University of Science and Technology, School of Medicine and Dentistry, Kumasi, Ghana; 3 Devices/Tools Global Program, PATH, Seattle, Washington, United States of America; Tulane University School of Public Health and Tropical Medicine, UNITED STATES

## Abstract

**Introduction:**

Preterm infants make up the majority of the 9 million babies born in Africa and South Asia requiring supplemental feedings as they transition to exclusive breastfeeding. The World Health Organization recommends the use of a cup to feed newborns with breastfeeding difficulties in low-resource settings. We set out to evaluate the Nifty cup, a new feeding cup designed specifically for infants with breastfeeding difficulties.

**Materials and methods:**

We conducted a randomized clinical trial in Ghana. We hypothesized infants would prefer the Nifty cup and that it would have less spillage as compared to a medicine cup. We enrolled mothers and preterm infants with breastfeeding difficulties indicated to cup feed at Komfo Anokye Teaching Hospital. Each mother-infant pair used the Nifty cup and a standard medicine cup; and two feeding assessments with each cup were conducted. We employed an intent-to-treat analysis comparing cup preference using a Wilcoxon signed rank test and spillage using generalized estimating equations.

**Results:**

We enrolled 200 mothers and 237 infants. Many infants were very low birth weight (62%), less than two weeks old (62%), and multiple birth (29%). In response to separate questions about each cup, more mothers reported liking the Nifty cup a lot as compared to the medicine cup (85% versus 57%, p<0.001). When asked to choose between the two cups, more than 75% preferred the Nifty cup (p < 0.001). There was slightly less spillage with the Nifty cup (8.9%) versus the medicine cup (9.3%), which was not statistically significant (p = 0.35). Mothers reported greater confidence and ease of using the Nifty cup and greater use one-month post-discharge compared to the medicine cup (p-values <0.001). Nearly all mothers were breastfeeding and cup feeding their infants at study initiation and at one-month post-discharge.

**Discussion:**

This is the first randomized clinical trial of cup feeding in sub-Saharan Africa. Mothers prefer the Nifty cup to a medicine cup for supplemental feeds to their preterm infant. The Nifty cup was used with greater ease and confidence. The Nifty cup can offer an improved feeding experience for the mother-infant pair.

## Introduction

Each year, approximately 9 million babies born in Africa and South Asia have difficulties breastfeeding because they are preterm, have craniofacial anomalies, or were born to mothers who died of childbirth-related causes [1–5]. Scaling up breastfeeding to a near universal level could prevent 823,000 deaths annually of children younger than five years old [6]. Preterm infants make up a substantial proportion of infants with breastfeeding difficulties and neonatal deaths [7–9]. Preterm infants developing their suck-swallow-breathe mechanism often require short-term supplemental feedings until they can transition to exclusive breastfeeding [10]. Feeding tools used in high-resource settings for infants with breastfeeding difficulties such as nasogastric tubes, specialized bottles, and breast pumps are impractical and unhygienic in settings that lack clean water and electricity [11].

The World Health Organization and United Nations Children’s Fund recommend hand expression of breast milk and the use of a small cup to feed newborns with breastfeeding difficulties in low-resource settings [12–14]. The body of evidence supporting cup feeding is growing [15–17]. A systematic review of studies from middle- and high-income countries concluded that feeding infants by cup is safe, although acceptability and compliance may be more challenging in high-income settings [17]. A different systematic review showed that cup feeding preterm infants resulted in improved physiology and higher short-term and long-term breastfeeding rates after discharge [15].

Cups are more easily sanitized than bottles and only require that the infant be able to swallow and breathe, which demands less energy and skill than the more complex suck-swallow-breathe mechanism needed for breastfeeding [18]. Yet, no standard cup for feeding infants with breastfeeding difficulties exists. Non-specific cups such as small shot glasses, bottle tops, medicine cups, paladai (a 10-mL beaked cup used in south India) [17], and generic drinking cups are used commonly along with spoons and syringes. Cups made of metal or hard plastic can cut the thin skin of the oral commissure of the preterm infant, thereby increasing risk of infection. Moreover, conventional cups are too small for hand expression of breast milk, which means breast milk is transferred from other, possibly contaminated containers prior to feeding to the newborn.

Most infant feeding cups have relatively wide rims. The wide rims on generic cups make it difficult for the inexperienced cup feeder, typically a new mother, to control the rate of milk flow to a newborn’s mouth. A cycle of poor feeding may quickly undermine a mother’s confidence in her ability to feed and care for her preterm infant. Additionally, rapid or inconsistent rate of milk flow from wide-rimmed cups results in a subpar feeding [19]. Improper flow rate results in spillage, stresses the infant, and can cause the infant to cough or aspirate, which in turn reduces essential nutritional intake and increases feeding duration and fatigue. Moreover, this also frustrates the mother, whose milk is laboriously hand expressed to only then be spilled and wasted. In one review, spillage was as much as 30% for every cup feed [17]. Over a short period of time, poor cup feeding may quickly result in chronic insufficient caloric intake that reduces endurance and increases a vulnerable neonate’s risk of undernutrition, failure to grow, infection, and death.

To address these problems with feeding cups, a team of investigators from Seattle Children’s Hospital, PATH, and the University of Washington designed the original Neonatal Intuitive Feeding TechnologY (NIFTY) cup. We employed an iterative user-centered process to create a reference design for an appropriate cup that could optimize intake and reduce feeding spillage, duration, and infection risk. We subsequently collaborated with Laerdal Global Health to refine and finalize the design, which resulted in the Nifty Feeding Cup by Laerdal [20] (the “Nifty cup”). The Nifty cup is a simple, easy-to-clean, ergonomic, and affordable tool designed to optimize feeding and the efficient hand expression of breast milk to newborn preterm infants and other infants with breastfeeding difficulties.

We propose the Nifty cup will improve the feeding experience of the mother-infant pair. The goal of this randomized crossover trial was to establish an evidence base for the Nifty cup by evaluating its effectiveness among 200 mothers and their preterm infants at a teaching hospital in Kumasi, Ghana. Our primary hypotheses were that the Nifty cup as compared to a standard medicine cup would be associated with greater caregiver satisfaction and less spillage.

## Materials and methods

We conducted a randomized clinical trial with a crossover component at Komfo Anokye Teaching Hospital (KATH) in Kumasi, Ghana, from August 2017 to September 2018. KATH is a large university and tertiary care hospital that has an estimated 9,000 births each year. It is a referral center for the central part of Ghana with a catchment area that spans six of the ten regions of the country.

### Participants

We enrolled caregivers with preterm infants with breastfeeding difficulties from the KATH in-hospital mother-baby unit. Eligibility required that the infant was born at KATH, at least 24 hours old, and less than 37 weeks gestational age at birth and at enrollment. Gestational age was estimated using a modified Dubowitz score [21]. We enrolled mothers with infants diagnosed with feeding difficulties who were clinically indicated to cup feed at the time of enrollment into the study. We restricted eligibility to infants who had an anticipated hospital stay of at least 48 hours to ensure all in-hospital data collection could be completed without incurring additional time or cost. Infants who were being fed using either a nasogastric tube or a cup were eligible. Infants with a major congenital anomaly (e.g., cleft palate) were not eligible, but those with a minor anomaly (e.g., hypoplastic fingernails or creased ear lobe) were eligible. A list of minor anomalies commonly observed that would not affect eligibility was compiled and used. For anomalies not on the list, research staff consulted with the pediatrician (author GPR) to determine if the anomaly was major or minor to determine eligibility. Infants with a condition that made them unlikely to comply with the study procedures (e.g., Down syndrome) were not eligible.

Eligibility required caregivers be willing to participate and more than 18 years of age at the time of the study. The caregiver had to be the mother or other person with primary responsibility for feeding the infant most of the time. The term “mother” is used to describe the caregiver.

### Intervention

**Fig 1:**
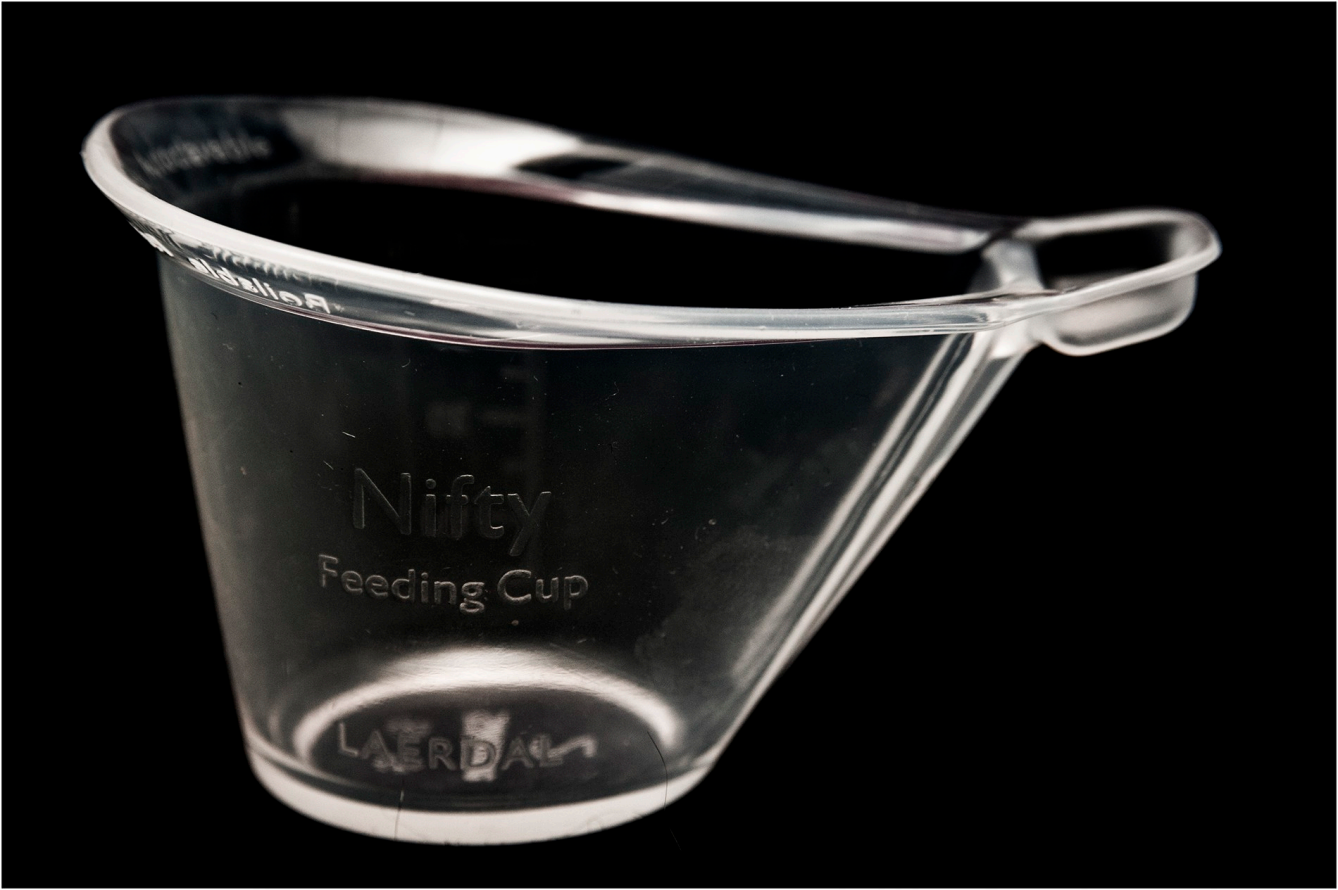
Nifty cup. Photo credit: Seattle Children’s/Erik Stuhaug.

Each mother-infant pair evaluated use of the Nifty cup and a standard medicine cup. The unique shape of the Nifty cup (Fig 1) aims to improve the mother’s experience with feeding, to enable the infant to participant in the feed, and to minimize spillage. The 40mL Nifty cup is large enough for direct hand expression of breast milk, and its soft material minimizes injury to the infant’s mouth and helps control flow. The Nifty Feeding Cup by Laerdal (Laerdal Global Health, Stavanger, Norway) is a reusable product made with silicone and can be stored in temperatures ranging from -40°C to +60°C. It can be cleaned by boiling in water for 10 minutes, steam autoclaving or chemical disinfection.

**Fig 2:**
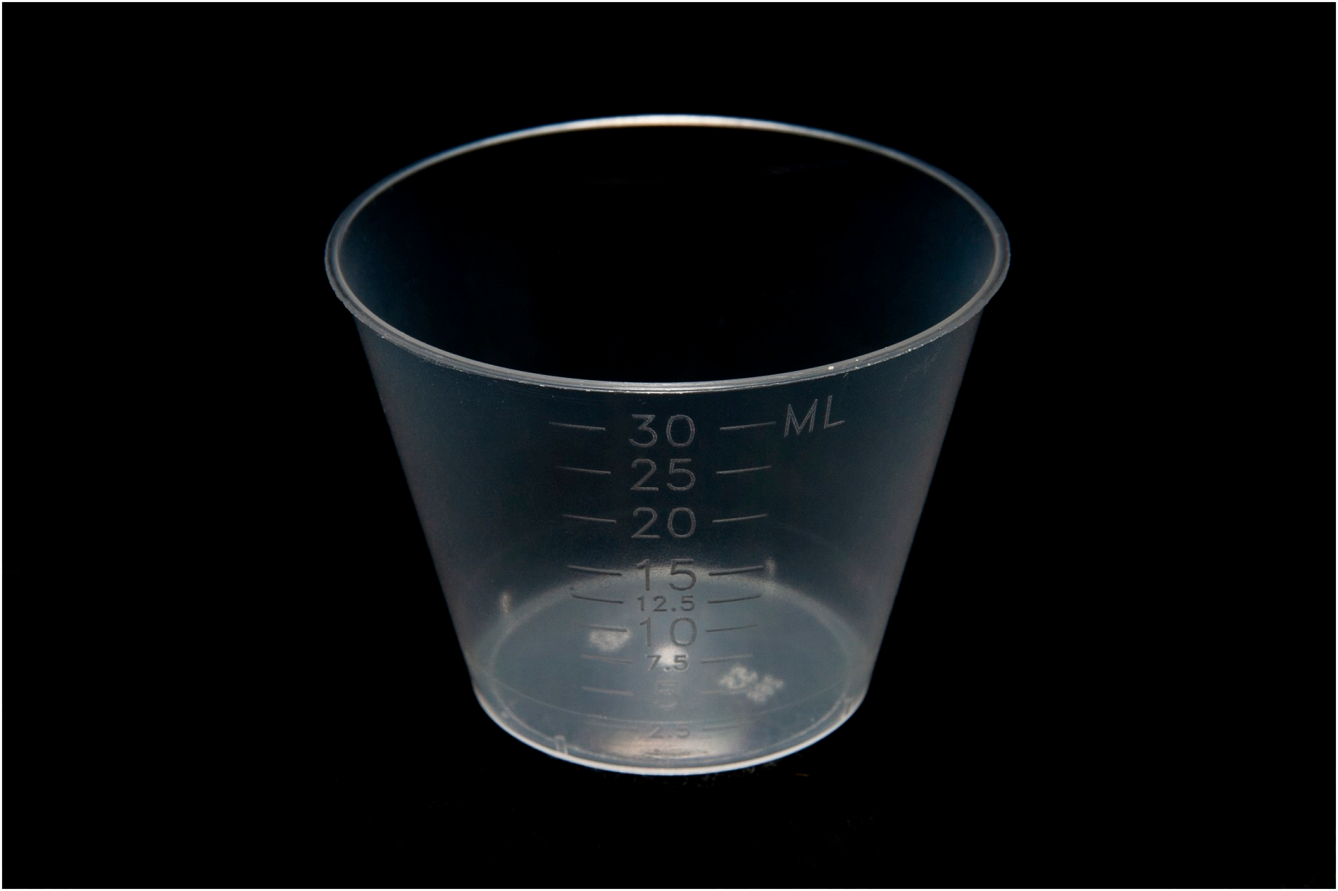
Medicine cup. Photo credit: Seattle Children’s/Erik Stuhaug.

A medicine cup—a standard 30 mL generic plastic medicine cup that is typically used to deliver medicines (Fig 2)—was used as the comparator. Small medicine cups are produced by a variety of manufacturers. Though they were not designed to feed neonates, they are commonly used in health facilities to feed breastmilk to infants who are having breastfeeding difficulties. The cups are generally translucent, calibrated with a variety of tick marks to indicate measurement from 2.5 to 30 mL.

### Randomization and blinding

The order of assignment was randomized using a random number generator in Microsoft Excel using randomly assigned block sizes of two and four to ensure concealment of the order of randomization. The randomization scheme was created at PATH (author PSC) before the start of the study. Sequentially numbered envelopes provided assignments according to the Excel spreadsheet. Immediately after the consent process was complete, the mothers were given the next envelope in the sequence by research staff. Nonclinical research staff were trained in the protocol and all assessments and administered all procedures including the randomization of the cup order and data collection from the study participants. The randomization scheme and creation and administration of the randomization envelopes was carried out by investigators or staff involved in the data collection or data analysis. It was not possible to blind the mother or the research staff to the cup assignment. The data analyst (author CMM) was blinded for all primary analyses.

### Procedures

Mothers used both the Nifty cup and the medicine cup to feed their infants. Consistent with a crossover design, the mother and infant were randomized to which intervention to receive first (Nifty or medicine cup). Randomization occurred after consent and the baseline survey was completed. The mother watched a short, two-minute video on how to use the assigned cup and was provided sufficient time to use the cup at least twice before the first feeding assessment. We conducted two feeding assessments with the first cup. The mother was then provided with the second cup, shown a short two-minute video on how to use that cup, and was provided sufficient time to use the second cup at least twice before the first feeding assessment. Two feeding assessments were conducted with the second cup. The mother then completed a preference survey comparing her experience with both cups. At four weeks post-discharge, a follow-up survey was conducted by telephone or in person for those who returned to clinic for their 1-month follow-up.

See S1 Table for study schematic detailing recruitment and study activities.

### Outcomes

We had two primary endpoints: preference and spillage. We hypothesized that mothers would prefer the Nifty cup over the medicine cup. To assess preference, we asked, after the mother had completed two feeding assessments with each cup, about her satisfaction with each cup. Responses were recorded on a five-point Likert scale ranging from “liked a lot” = 1 to “really did not like” = 5. Comparison of the Likert scale responses was our primary outcome. We also asked, in a single question, as to whether the mother preferred the Nifty cup or the medicine cup. This question was asked both in-hospital after all feeding assessments were completed and post hospital discharge.

The other primary endpoint was the amount of spillage. We hypothesized that the Nifty cup would have less spillage than a medicine cup. We measured spillage of milk by having the mother use a baby bib provided by the study to capture spilled milk and to wipe up milk from the infant’s mouth. The bibs used in the study were made of soft cotton cloth on the front and the backing had a plastic liner. Each bib measured approximately 20.8 x 23.8 cm. The bib was weighed and recorded in grams before and after each feeding assessment using a digital scale. The difference between the pre- and post-weight of the bib was used to measure the amount spilled. Because the amount spilled may vary by the amount prescribed, we estimated spillage as a percent, with the amount of milk in grams captured by the bib divided by the total amount of the milk fed to the infant. Total amount of the milk fed to the infant was measured by weighing the cup with milk before and after the feeding. Cup refills were also measured before and after the milk refill and the milk remaining at the end of the feed to generate a total amount of milk fed to the infant.

Pre-planned secondary analyses included examining the mother’s reporting of feeding experiences (confidence in using the cup, ease of measuring intake, hand expression) with each cup in-hospital and after hospital discharge, as well as breastfeeding, cup use and preference one month after hospital discharge. We also examined the duration of the feed and total intake. We considered a duration of feed greater than 20 minutes as a threshold above which the infant would be likely to expend more energy than that obtained from the milk. Staff measured duration of feeding in minutes and seconds with a digital timer. The timer was started at the time the cup first touched the infant’s lip and stopped when the cup last left the infant’s mouth. Stops and starts in the feeding (e.g., mother needed to express more milk) were documented and accounted for in our measure of duration of feeds. Intake was initially our primary outcome but because the provider prescribed the amount of milk/formula for each infant feed, we analyzed amount of intake solely as an exploratory question and made spillage and caregiver satisfaction our primary outcomes. This was done prior to starting the study.

### Analysis

We generated percentages, means, and standard deviations for infant and mother and household demographics and birth and feeding characteristics. To evaluate our hypotheses, we used an intent-to-treat analytic approach. We used a Wilcoxon signed rank test to compare the mother’s ranking of each cup (primary hypothesis) and feeding experiences across the two cups. A binomial test was used to evaluate the mother’s preference question in-hospital. and post-discharge comparing the preference to the presumption of equal preference between the two cups. We examined spillage (primary hypothesis) and other outcomes (feeding duration, amount) using generalized estimating equations (GEE) with robust standard errors. The same GEE approach was used to examine spillage by absolute amount (mL). The GEE models account for clustering within infants across feeding assessment with each cup. Because this is a single-site randomized clinical trial with an intent-to-treat analytic approach, no other covariates were included in our statistical models.

### Power

Power for this study was determined by our hypothesis on spillage. With 200 pairs, we estimated we would be able to detect at least a 5% difference in spillage and preference with 90% power and an alpha of 0.05 if no carryover effects were present. Carryover effects occur when a participant learns from cup feeding with the first cup, which then affects the outcomes when feeding with the second cup. If carryover effects were observed, we would have 80% power to detect an average 5% difference. This difference is conservative given studies of reported differences in spillage are between 6.3 and 7.8 ml [22–24]. We used a published estimate of a cup feeding standard deviation of 11 [22] and conservative correlations ranging from 0.02 to 0.50 when estimating power. We used the “power” command in Stata 13.0 to estimate power using a two-sided paired t-test.

To assess for potential carryover effects, we used stratified analyses to evaluate statistically significant differences by cup type and feeding assessment number. We tested interaction terms using a threshold p-value of < 0.05 to indicate the presence of carryover effects. This was assessed for spillage, duration of feeding, and intake since these were measured across multiple feedings. We did not evaluate carryover effects for preference since this was assessed at one point in time. To assess for potential confounding by feeding factors, we evaluated a range of feeding characteristics by cup type (e.g., source of nutrition, amount recommended to feed, number of feeds since prior visit).

This study was reviewed and approved by the PATH Research Ethics Committee on May 11, 2017and the KATH Committee on Human Research, Publications and Ethics of Kwame Nkrumah University of Science and Technology (KNUST) on May 24, 2017. Written informed consent was obtained from the mothers for their participation and the participation of their infant in this study. Participants were able to keep both feeding cups evaluated and the bib used to measure spillage and were provided with five diapers for their time and effort. Interactions between study staff and participants, including the reading of the consent form, were in English or Twi at the preference of the mother. The authors confirm that all ongoing and related trials for this intervention are registered. We registered the trial on ClinicalTrials.gov (#978912–3) on September 6, 2017. Patient recruitment and follow-up was conducted from August 8, 2017 to September 25, 2018. The delay in registering this study one month after enrolment of participants started was due to travel and coordination efforts across sites and was in compliance with trial registry guidance. We did not look at any data during this period. We posted the study protocol on ClinicalTrials.gov on January 24, 2019.

## Results

**Fig 3:**
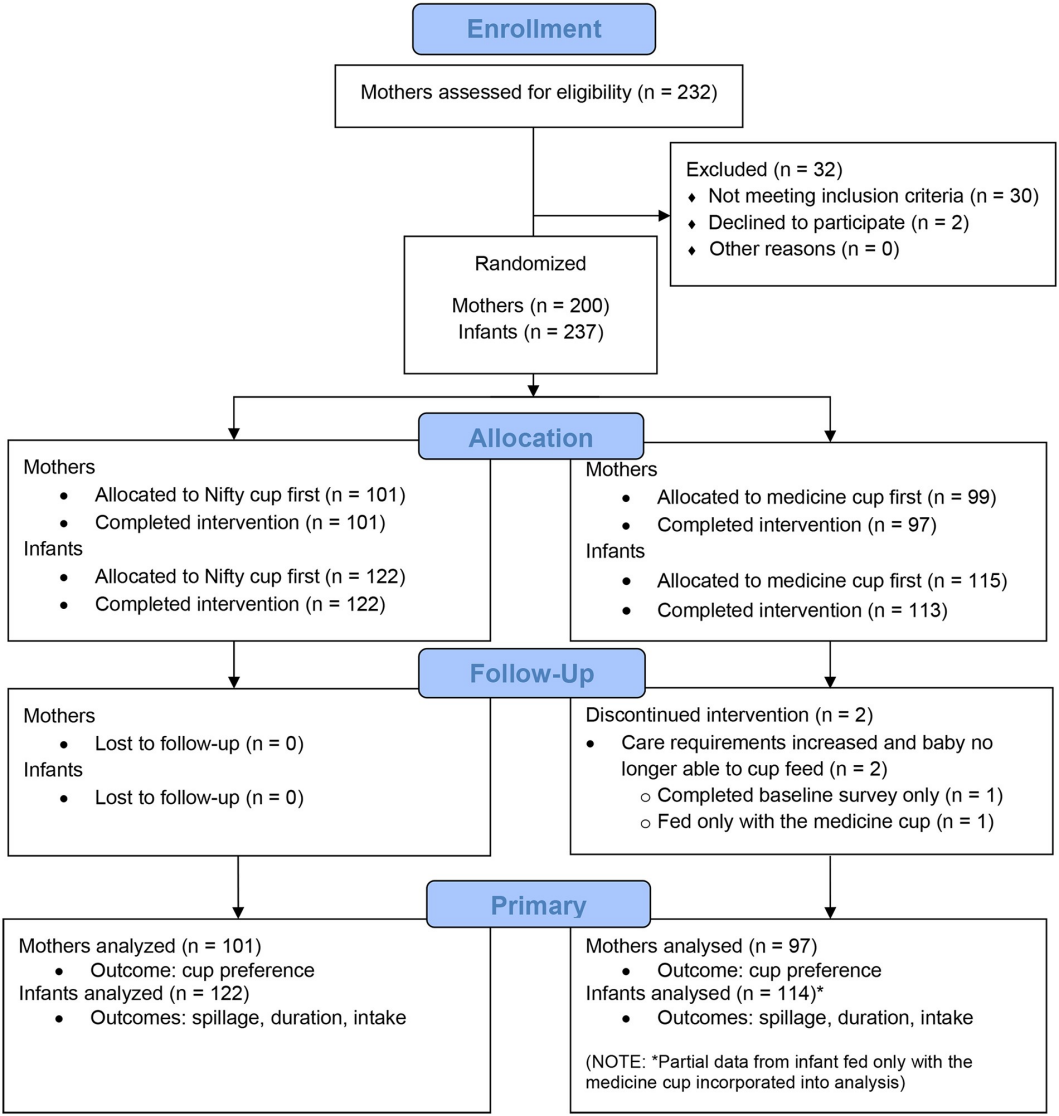
CONSORT flow diagram.

Our consort flow diagram outlines eligibility assessment, allocation, follow-up, and analysis (Fig 3). We enrolled 200 mothers and 237 infants with high levels of compliance throughout the study. Among those enrolled, 198 mothers and 236 infants were included in analyses of our primary outcomes Just under half were male (Table 1). Of the infants enrolled, 169 were singletons, 50 were twins, and 18 were triplets. Nearly two-thirds of the infants were 1,000–1,500 grams at birth and half were one to two weeks old at enrollment (Table 1).

**Table 1:** Baseline characteristics of infants.

	All combined	
	(N = 237)	%
Infant sex, %		
Male	110	46.4
Female	127	53.6
Baby’s age at enrollment (in days), %		
< 1 week	18	8.8
1–2 weeks	108	52.7
2–3 weeks	40	19.5
≥ 3 weeks	37	19.0
Birth weight (in grams), %		
Extremely low birth weight (< 1,000)	24	10.1
Very low birth weight (1,000–1,500)	147	62.0
Low birth weight (1,500–2,500)	66	27.9
Gestational age at birth (in weeks) mean (sd)	237	30.2 (2.3)
Corrected gestational age at enrollment (in weeks), mean (sd)	237	31.2 (1.9)
Multiple birth status, %		
Singleton	169	71.3
Twin	50	21.1
Triplet	18	7.6

All but one caregiver was the infant’s mother (Table 2). The mean age of mothers was 28.5 years old. The majority of mothers had a highest level of education at the primary or junior high school level. More than 80% of participants were married, and more than 90% had a cellphone and lived in a house with electricity. For more than 40% of the participants, the infant enrolled was the mother’s first live birth. Nearly all had breastfed their infants and were currently breastfeeding and cup feeding their infants. More than 80% were using the top of a bottle to cup feed their infants at baseline and more than two-thirds of the infants had been tube fed (Table 2).

**Table 2:** Demographics and mother’s birth and feeding characteristics^A^.

Mother and household demographics	(N = 200)	% / mean (sd)
Mother was caregiver type, %	199	99.5
Mother’s age, mean (sd)	200	28.5 (6.0)
Mother’s highest level of education completed, %		
Primary / no formal schooling	30	15.0
Junior high school	81	40.5
Secondary	42	21.0
Technical	12	6.0
University	35	17.5
Mother is married, %	166	83.0
Mother worked in past year, %	129	64.5
Mother has mobile phone, %	194	97.0
Number of individuals in household, mean (sd)	199	3.8 (1.8)
Home has electricity, %	184	92.0
Adult sleeping platform, %		
Bed with mattress	179	90.0
A mat on the floor/other	20	10.0
**Birth and feeding characteristics**^B^		
Number of prior live births, %		
0	85	42.7
1	37	18.6
2	26	13.1
3	24	12.1
4+ (max = 7)	27	13.6
Caesarean section, %	91	45.5
Ever breastfed baby, %	199	100.0
Currently breastfeeding baby, %	198	99.5
Currently cup feeding baby, %	196	98.0
Cup fed baby ≥ 12 times in past day, %	160	82.9
How active baby suckling when breastfeeding, %		
Very active	140	70.7
Somewhat active	31	15.7
A little / not active	27	13.6
Cup used to feed baby (pre-enrollment), %		
Top of a bottle	163	83.2
Ghana cup	27	13.8
Syringe / other	6	3.1
Baby ever been tube fed, %	141	70.5

^A^Variable missing less than 3% unless otherwise noted.

^B^Mother queried once on feeding characteristics even if she had twins/triplets.

**Fig 4:**
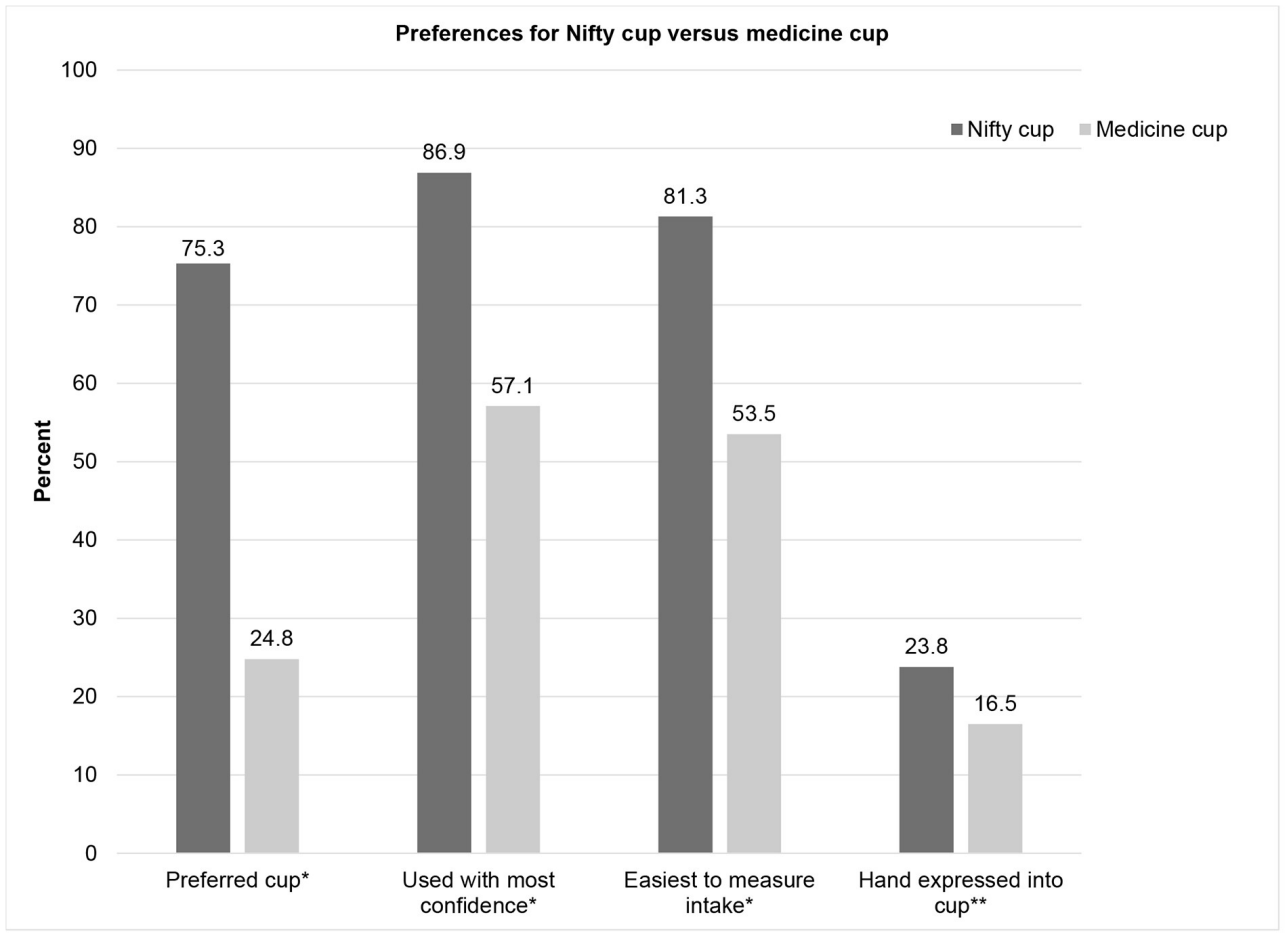
Preferences for Nifty cup versus medicine cup. *p < 0.001, **p < 0.01, among the 177 who reported hand expressing at baseline.

There was a clear preference for the Nifty cup over the medicine cup. On the Likert scale questions regarding how much they liked each cup, 85% liked the Nifty cup a lot compared to just over half reporting they liked the medicine cup a lot (p < 0.0001) (Table 3). When asked to choose between the two cups, more than 75.3% preferred the Nifty cup; the preference ratio (75.3% with a Nifty cup preference to 24.7% with a medicine cup preference) was statistically different than our test of no difference (50%:50% ratio) (0.32, 95% CI 0.25, 0.41, p < 0.001, Fig 4). Metrics that evaluated feeding preferences showed a preference for the Nifty cup. More than 80% had the greatest confidence in feeding with the Nifty cup, and more than 80% found it easier to measure intake (both p < 0.001). More mothers hand expressed into the Nifty cup (23.8%) than the medicine cup (16.5%) (p < 0.01).

**Table 3:** Outcomes (All analyses N = 236 infants, 198 mothers).

	Nifty cup	Medicine cup	Difference (95% CI)	p-value
	Estimate	Estimate		
Cup preferred, median (IQR)	1 (1,1)	1 (1,2)		
Cup preferred, %				< 0.0001
Liked a lot	84.9	57.1		
Okay	12.6	32.8		
Did not like / neutral	2.5	10.1		
Spillage, % of feed provided (mean, sd)	8.9 (6.7)	9.3 (8.7)	0.4 (1.3, -0.5)	0.35
Duration of feeding > 20 minutes, %	31.5	28.6	-2.9 (-7.9, 2.2)	0.26
Intake, mL (mean, sd)	16.6 (8.2)	16.6 (7.8)	0.02 (0.59, -0.53)	0.92

We conducted stratified analysis by gestational age at enrollment and by birthweight among mother-infant dyads (N = 198) and found no differences in cup preference by these variables. Among dyads with infants <32 weeks gestational age at enrollment, 71.9% preferred the Nifty cup versus 77.6% of infants ≥32 weeks, when asked to choose between the two cups (p = 0.37). Likewise, 82.6%, 73.3%, and 76.4% of dyads with infant birthweights <1000 grams, 1000–1499 grams and 1500 grams and above respectively, preferred the Nifty cup to the medicine cup when asked to choose between the two cups (p = 0.62).

There was 0.4% less spillage with the Nifty cup compared to a medicine cup, with a 95% CI ranging from 1.3% less spillage to 0.5% more spillage. There was no difference in our secondary outcomes (intake and duration of feeding of greater than 20 minutes) (Table 3).

At one-month hospital discharge, 99% of mothers reported currently breastfeeding and 99% reported having used a feeding device since hospital discharge to feed their babies (Table 4). Of those who had used a device, 70% used the Nifty cup as their primary feeding tool. Nifty cup users reported they preferred the cup because there was less spillage (83%), it was easy and convenient (86%), and it was safe to use (80%) (Table 4).

**Table 4:** Feeding status post hospital discharge (N = 196).

	%
Days since discharge (mean, sd)	26.2 (3.7)
Currently breastfeeding, %	98.9
Used a device to feed baby since discharge, %	98.9
Preferred device^1^, %	
Nifty	71.4
Medicine cup	28.1
Other	0.5
Reasons for preferred use of Nifty post discharge, %	
Less spillage	82.6
Easy and convenient to use	85.6
Safe	79.6
Liked the material	52.3
Not stressful	35.6

^1^p-value < 0.02.

In evaluating potential carryover effects, we observed no statistically significant interaction between cup and feeding assessments on any of our outcomes, indicating no carryover effects related to our crossover approach (S2 Table). In our evaluation of feeding characteristics by cup type, we found no statistically significant differences, which suggests randomization produced groups whose feeding characteristics were similar for the two cups (S3 Table). There were no unanticipated harms or effects using either cup.

## Discussion

To our knowledge, this is the first randomized clinical trial of cup feeding in sub-Saharan Africa and the first clinical trial of the Nifty cup in any country. The results from this study show that mothers of preterm infants preferred the Nifty cup compared to a medicine cup to provide supplemental feedings both in-hospital and post-discharge. Mothers preferred the Nifty to the medicine cup because it was easier to use and because they had greater confidence in their ability to feed their babies with the Nifty cup when compared to the medicine cup. Almost all the mothers were breastfeeding and cup feeding their infants prior to the start of the study. Most of the mothers reported cup feeding with the top of a bottle, which more closely resembles a medicine cup. The familiarity of mothers with using the top of a bottle to cup feed did not seem to affect their cup preference. More mothers hand expressed into the Nifty cup. There was a little less spillage with the Nifty versus the medicine cup, but this difference was not statistically significant.

The overall high acceptability of the Nifty cup for feeding preterm infants means the Nifty cup can play a pivotal role in sustaining evidence-based care practices for preterm infants such as kangaroo mother care [25]. Though no global standard definition of kangaroo mother care currently [26] exists, components generally consist of continuous skin-to-skin contact to ensure thermostability and other health benefits to the infant, support for exclusive breastfeeding or other appropriate feeding, and early recognition or response to illness and/or discharge when indicated [27]. In the case of preterm infants, exclusive breastfeeding is often not achieved at birth, and infants require ongoing supplemental feeding while they mature and develop the skills to transition to exclusive breastfeeding. The Nifty cup fits into the existing paradigm for kangaroo mother care by facilitating exclusive breastfeeding. The Nifty cup also fits with the World Health Organization recommendations for supplemental feeding [12,28].

Mothers’ reported high acceptability and confidence in using the Nifty cup indicates that it can play a critical role in facilitating the transition to exclusive breastfeeding. A systematic review of 103 publications related to barriers and enablers of mother care practice identified “breastmilk expression and other breastfeeding-related issues” as being in the top ten barriers to adoption of breastfeeding for women in low- and middle-income countries [29]. Further, this same review showed that “feelings of confidence / empowerment” was one of the top five enablers for women in low- and middle-income countries and elsewhere [29]. That mothers reported the cup was easy to use and that they had confidence in using the cup to feed their infants suggests the Nifty cup may serve as a confidence enabler for mothers with preterm infants. Providing confidence-building tools to mothers with vulnerable infants in low- and middle-income countries can help them provide high-quality care for their preterm infant both during their hospital stay and after discharge.

There are several potential explanations for our finding of no statistically significant difference in spillage between the two cups. There may be no difference in spillage between the two cups or the difference is so small as not to be clinically relevant. However, given more than 80% of mothers reported that reduced spillage was a key reason they liked the Nifty cup, it may be that we were unable to adequately measure spillage. Spillage often occurs in the form of dribble down from the edge of the mouth and down the neck, so that it may not be caught by the bib, and to the extent this was not captured by the cups, our measurement could be inaccurate. It may also be that mothers in this study were better cup feeders than in the general population as they received guidance on cup feeding from the provider, our research staff, and via our video. Alternatively, the experience of being watched by study staff at feeding assessments may have made mothers more careful in their feeding of their infants with both cups, thereby reducing spillage and not providing an accurate picture of what typically occurs during feedings. Spillage in this study was low for both cups at less than 10% per feed. The spillage proportion reported in this study which is similar to reports using the paladai (a cup widely used in South India) [17], which studies report as 6% to 12% [24,30], however it is substantially lower than for other cups at 25% to 30% [23,24].

Though hand expression was not discussed or the focus of our intervention, it is notable that more participants reported hand expressing breastmilk into the Nifty cup than the medicine cup. This is most likely due to the design and larger volume size of the Nifty cup, which was optimized ergonomically for use during hand expression so that mothers would not need to transfer their expressed breastmilk to another container to feed their infants and would have the ability to express enough milk for the next feed. Though a Cochrane review found no association between hand expression or pump and bacterial contamination [31], their finding was based on one study in the United States and one study in Malaysia. The study in Malaysia found a statistically significant increase in bacterial contamination with a pump and not hand expression in the home setting but no difference in hospital settings [32]. Given many mothers in low-resource settings are discharged from hospital while still providing supplemental feeds, tools that support hand expression such as the Nifty cup could be useful. Evidence also currently suggests hand expression can be a powerful tool, particularly in the context of relaxation, to facilitate breast milk supply and the transition to exclusive breastfeeding [31]. Thus, the greater use of the Nifty cup for hand expression may indicate that mothers found its use allowed for a more relaxed and positive expression of the feeding experience.

We were not surprised that we did not observe differences in intake or duration of feeding. Mothers were given medical advice by hospital physicians overseeing their care prescribing how much to feed their infants at each feeding as well as advice on feeding duration. So, it was not our expectation that there would be much, if any, difference in intake or feeding duration between the two cups. We were not aware of the proscriptive nature of the amount of feeding until just before the study started and so spillage was quickly moved to be a primary outcome alongside caregiver preference. Additionally, we were not powered to observe differences in the proportion of infants fed for longer than 20 minutes and no such difference were observed.

We found no statistically significant interactions between feeding assessment number and cup type on spillage or our other measures across feedings. This is reassuring as it suggests that the feeding differences did not depend on feeding assessments over time. It is also reassuring that differences in the feeding characteristics across the two cups were not statistically significant different. This indicates our findings were not influenced by factors other than the cup.

This study has several strengths. To our knowledge this is the first randomized clinical trial of cup feeding in sub-Saharan Africa and the first research on the Nifty cup in Africa. Our findings are likely to be relevant to millions of infants born preterm in Africa and other low- and middle-income countries who need supplemental feeds while they transition to exclusive breastfeeding. Participants enrolled in our study were compliant and were retained at a very high rate. That spillage was below 10% provides additional support for cup feeding as a safe and viable method of feeding preterm infants. Most studies of cup feeding have been conducted in high-resource settings in which the feeder is a nurse or lactation specialist. Our study evaluated mothers cup feeding, which is a more “real-world” experience in most low- and middle-income settings. Our data suggest that mothers can be effective feeders of preterm infants and that cup preference may play an important enabling role in confidence building for caring for a preterm infant.

Our work also has limitations. Using bib weights to capture spillage may not be as accurate as we had anticipated. This is speculation based on the vast majority of mothers reporting they liked the Nifty cup because it prevented spillage. This suggests that the method to collect spillage data could be improved and that the amount of spillage perceived by mothers may be the more important variable in terms of product uptake and consistent use. Nevertheless, the low level of spillage overall suggests that cup feeding can be successfully managed by mothers. Efforts to minimize long feeding times through education and monitoring may be beneficial given that more than a quarter of participants had feeding times that exceeded 20 minutes.

Mother-infant dyads enrolled were all in-patients in the KATH mother-baby unit. This was an open room with between 9 and 13 beds, and mothers could see the different types of cups being used and talked among themselves about the different cup types. This could have influenced their perspectives about the cups, although the short time frame of the study and patient length of stay most likely did not allow for formation of a strong group opinion. We lacked sufficient time and funding to look at outcomes such as weight gain over time and time to exclusive breastfeeding.

## Conclusion

This is the first randomized clinical trial of cup feeding in sub-Saharan Africa. Mothers prefer the Nifty cup over a medicine cup to feed their preterm infant. The Nifty cup was used with greater ease and confidence and mothers were more likely to hand express into the Nifty cup. A small non-statistically significant difference was observed for spillage. Our findings show that the Nifty cup can offer an improved feeding experience for the mother-infant pair.

## Supporting information

S1 Table Study procedures. (DOCX)

S2 Table Evaluating carryover effects by assessing the impact of feeding assessments over time by cup on outcomes. ^1^Compare cups for feeding assessments 1 and 2; ^2^Compares cups for feeding assessments 3 and 4; ^3^Evaluates the interaction between cup type and feeding assessment number on outcome.
(XLSX)

S3 Table Evaluating potential confounding by time and varying feeding characteristics by cup. (XLSX)

S1 Appendix S1 Appendix_08_Feeding_Assessment_Ver 4_24apr17.docx. (DOCX)

S2 Appendix S2 Appendix_10_In Hospital Preference Survey_Ver 4_21apr17.docx. (DOCX)

S3 Appendix S3 Appendix_12_Follow_Up_Survey_Ver 3_16apr17.docx. (DOCX)

S4 Appendix S4 Appendix_13_Protocol_ver 7_24jan19.docx. (DOCX)

S5 Appendix S5 Appendix_14_CONSORT checklist_24jan19.docx. (DOCX)
