# Supplementary material for: A randomized crossover trial comparing the Nifty cup to a medicine cup in preterm infants who have difficulty breastfeeding at Komfo Anokye Teaching Hospital (KATH) in Kumasi, Ghana
Source: PLoS One. 2019 Oct 17;14(10):e0223951. doi: 10.1371/journal.pone.0223951 (PMC6797128; doi:10.1371/journal.pone.0223951)
Supplement: S1 Table — (DOCX) [file pone.0223951.s001.docx]

**S1 Table—Study Procedures.**

| Pre-screening  Day –7 to 0 | - Identify potentials from (1) chart, (2) provider - Provider introduces study to caregiver/mother |
| --- | --- |
| Visit 1  Eligibility and consent | - Study introduction and overview - Screen mother for eligibility - Consent process |
| Baseline  Day 1, Hour 0 | - Random assignment to Cup 1 - Baseline survey - Provide Cup 1 + video + instructions for use |
| Visit 2  Cup 1, Feed 1  > 4 hours after Visit 1 | - Complete feeding assessment |
| Visit 3  Cup 1, Feed 2  > 2 hours after Visit 2 | - Complete feeding assessment - Remove Cup 1 - Provide Cup 2 + video + instructions for use |
| Visit 4  Cup 2, Feed 1  > 4 hours after Visit 3 | - Complete feeding assessment |
| Visit 5  Cup 2, Feed 2  > 2 hours after Visit 4 | - Complete feeding assessment - Complete in-hospital preference survey - Return Cup 1 to mother - Provide thank you (5 diapers) - Schedule follow-up survey |
| Visit 6  Post-discharge  4 weeks post-discharge | - Conduct follow-up survey 4 weeks post-discharge - Thank the participant verbally once more |
