# Supplementary material for: A randomized crossover trial comparing the Nifty cup to a medicine cup in preterm infants who have difficulty breastfeeding at Komfo Anokye Teaching Hospital (KATH) in Kumasi, Ghana
Source: PLoS One. 2019 Oct 17;14(10):e0223951. doi: 10.1371/journal.pone.0223951 (PMC6797128; doi:10.1371/journal.pone.0223951)
Supplement: S1 Appendix — (DOCX) [file pone.0223951.s004.docx]

Feeding Assessment, **v4, 24April17**

**NEXT VISIT @:** _____ _____:______

Day Time

**STUDY ID #__________ STAFF INITIALS:__________DATE** _____/_____/_____**TIME:______:_______**

(day/month/year)

**Consented Caregiver:** ❑Mother ❑Grandmother ❑Aunt

THIS SECTION AND THIS FORM IS CRITICAL. BE SURE EVERY PART OF THIS FORM IS FILLED OUT IN FULL.

1. **FEEDING CHARACTERISTICS**

|  | **RA Set Up Details** |  | |
| --- | --- | --- | --- |
| 1. | Feeding Assessment Number | ❑1  ❑2  ❑3  ❑4 | |
| 2. | Cup used in Feeding Assessment | ❑Nifty  ❑Medicine cup | |
|  |  |  | |
| 3. | Feeder Is:  [make sure same as consented caregiver] | ❑Mother  ❑Grandmother  ❑aunt | |
|  |  |  | |
|  | **Questions for the Feeder. (Answer before or after feed)** | | |
| 4. | What is your baby being fed? | ❑Breast Milk  ❑Formula  ❑Other | |
|  |  |  | |
| 5. | How much does your health care provider currently suggest you feed your baby? | \|_______________\| Grams | |
| 6. | Since my prior visit at ____:_____[& DATE], how many times have you fed your baby?  [HELP MOM ESTIMATE BASED ON TIME INTERVAL OF FEEDS. ASK IF THEY HAVE MISSED ANY FEEDS AND SUM.] | ________________________________ | |
| 7. | Since my prior visit, what feeding methods have you used to feed your baby?  READ ALOUD. CHECK ALL THAT APPLY | ❑Breast feed only  ❑Cup feed only  ❑Cup and breast  ❑Other, describe:________________ | |
| 8. | List the method used most, then next most and so on. And then tell us about how many feeds with each method.  GUIDE MOTHER. ALL METHODS MUST ADD UP TO NUMBER OF FEEDS REPORTED IN QUESTION 6 | Method | No. of Feeds |
|  |  | 1. |  |
|  |  | 2. |  |
|  |  | 3. |  |
|  |  | 4. |  |
|  |  | total # of feeds (see Q6) | _______ |
| 9. | Since my last visit, what types of feeding cups have you used to feed your baby?  CHECK ALL THAT APPLY | ❑Medicine Cup  ❑Nifty cup  ❑Ghana Cup  ❑Bottle Top  ❑Other, describe:______________ | |
| 10. | List the cup used most, then next most and so on. And then tell us about how many feeds with each cup.  ALL METHODS MUST ADD UP TO NUMBER OF FEEDS REPORTED IN QUESTION 6 | Type of cup | No. of Feeds |
|  |  | 1. |  |
|  |  | 2. |  |
|  |  | 3. |  |
|  |  | 4. |  |
|  |  | total # of feeds (see Q6) | _______ |
| 11. | At these feedings, how much did you feed your baby? [READ ANSWERS ALOUD.] | ❑The recommended amount  ❑Less than the recommended amount  ❑More than the recommended amount  ❑Don’t know | |
| 12. | Since my prior visit, how many times have you expressed breast milk? | ________________________________ | |
| 13. | At what time was the cup feeding just before this one? | _______:_______  TIME | |
| 14. | At the cup feeding just before this one, how much did you feed your baby? | ❑The recommended amount  ❑Less than the recommended amount  ❑More than the recommended amount  ❑Don’t know | |
| 15. | At what time was the breastfeeding just before this feeding? | _______:_______  TIME | |
| 16. | How many minutes did this breastfeeding last? | _______:_______  Minutes | |

1. **INITIAL BIB WEIGHT, MILK AMOUNTS AND FEEDING TIMES**

B5

B1

B4

B3

B2

| **START** | **END** | |
| --- | --- | --- |
| **START BIB WEIGHT** | **END BIB WEIGHT** | |
| \|  \| Grams \| \| --- \| --- \| | \|  \| Grams \| \| --- \| --- \| | |
| ❑Not measured, reason:________________ | ❑Not measured, reason:________________ | |
| **START VOLUME IN CUP** | **END VOLUME IN CUP** | |
|  | | |
| VOLUME:   \|  \| mL \| \| --- \| --- \| | VOLUME:   \|  \| mL \| \| --- \| --- \| | |
|  |  | |
|  |  | |
| ❑Not measured, reason:________________ | ❑Not measured, reason:________________ | |
| **START WEIGHT IN CUP** | **END WEIGHT IN CUP** | |
| WEIGHT:   \|  \| Grams \| \| --- \| --- \| | WEIGHT:   \|  \| Grams \| \| --- \| --- \| | |
|  |  | |
| ❑Not measured, reason:________________ | ❑Not measured, reason:________________ | |
| **START TIME** | **END TIME** | |
| FEED START TIME _____:_____:_____  (hour : minutes : seconds)  ❑Not measured, reason:________________ | FEED STOP TIME _____:_____:_____  (hour : minutes : seconds)  ❑Not measured, reason:________________ | |
| 1. WAS THE FEEDING STOPPED TO REFILL THE CUP? | | Yes ❑SKIP TO **SECTION C**  No ❑CONTINUE TO QUESTION 2 |
|  | |  |
| 2. WAS THE FEEDING TEMPORARILY STOPPED WITHOUT REFILLING? | | Yes ❑GO TO **SECTION B5**  No ❑CONTINUE TO **SECTION D** |
| **TEMPORARY STOP (NO REFILL)** | | |
| Reason stopped feeding without refill:___________________________ | | |
|  |  | |
| **START TIME AFTER STOP** | **END TIME AFTER STOP** | |
| FEED RESTART Time _____:_____:_____  (hour : minutes : seconds)  ❑Not measured, reason:______________  __  *THIS SECTION NOT IN REDCAP* | FEED STOP Time _____:_____:_____  (hour : minutes : seconds)  ❑Not measured, reason:________________  **COUNT (TALLY) TIMES DURING WHOLE FEED (ADD TO SECTION G1-6)**   \| COUGH \|  \| BREATHING \|  \| \| --- \| --- \| --- \| --- \| \| SPIT-UP \|  \| LYING DOWN \|  \| \| DRIBBLE \|  \| POURING \|  \| | |

COMPLETE DURING ENTIRE FEED

**SECTION C. REFILL MILK AMOUNTS AND FEEDING TIMES**

C6

C5

C3

C2

C4

C1

| **Was the cup refilled?** | ❑ YES  ❑ NO SKIP TO SECTION D |
| --- | --- |
| **START** | **END** |
| **START VOLUME IN CUP** | **END VOLUME IN CUP** |
|  | |
| VOLUME:   \|  \| mL \| \| --- \| --- \| | VOLUME:   \|  \| mL \| \| --- \| --- \| |
|  |  |
|  |  |
| ❑Not measured, reason:________________ | ❑Not measured, reason:________________ |
| **START WEIGHT IN CUP** | **END WEIGHT IN CUP** |
| WEIGHT:   \|  \| Grams \| \| --- \| --- \| | WEIGHT:   \|  \| Grams \| \| --- \| --- \| |
|  |  |
| ❑Not measured, reason:________________ | ❑Not measured, reason:________________ |
| **START TIME** | **END TIME** |
| REFILL START Time _____:_____:_____  (hour : minutes : seconds)  ❑Not measured, reason:________________ | REFILL STOP Time _____:_____:_____  (hour : minutes : seconds)  ❑Not measured, reason:________________ |
| 1. WAS THE FEEDING STOPPED TO REFILL THE CUP? | Yes ❑COMPLETE ADDITIONAL PAGE OF **SECTION C**  No ❑CONTINUE TO **QUESTION 2** |
|  |  |
| 2. WAS THE FEEDING TEMPORARILY STOPPED WITHOUT REFILLING? | Yes ❑GO TO **SECTION C6**  No ❑CONTINUE TO **SECTION D** |
| **TEMPORARY STOP (NO REFILL)** |  |
| REASON STOPPED FEEDING WITHOUT REFILL: | ____________________________________ |
| **START TIME AFTER STOP** | **END TIME AFTER STOP** |
| REFILL RESTART Time _____:_____:_____  (hour : minutes : seconds)  ❑Not measured, reason:________________ | REFILL STOP Time _____:_____:_____  (hour : minutes : seconds)  ❑Not measured, reason:________________ |

IF ADDITIONAL BREAKS IN FEEDING WERE NEEDED, PLEASE USE AN ADDITIONAL PAGE OF SECTION C.

**SECTION D. POST FEED BIB WEIGHT AND ASSESSMENT**

|  | WIPE ANY REMAINING MILK FROM MOUTH, NECK, AND FACE BEFORE MEASURING. | | |
| --- | --- | --- | --- |
|  | 1. How much of the time did the baby actively try to control the rate of drinking? |  | All of the Time ❑  A lot of the time ❑  Some of the Time ❑  A little of the time ❑  Not at all ❑ |

**E. QUESTIONS FOR CAREGIVER**

Now we are going to ask you a few questions about your experience using this cup. READ RESPONSES ALOUD IF PARTICIPANT HAS DIFFICULTY ANSWERING.

| 1. | Overall how much did you like using this cup to feed your baby? |  | like a lot ❑  liked/ok ❑  neutral ❑  didn’t like ❑  really didn’t like ❑ |
| --- | --- | --- | --- |
|  |  |  |  |
| 2. | How much do you think your baby liked feeding with this cup? |  | like a lot ❑  liked/ok ❑  neutral ❑  didn’t like ❑  really didn’t like ❑ |
|  |  |  |  |
| 3. | How easy was it to feed your baby with this cup? |  | Very Easy ❑  Somewhat Easy ❑  Neutral ❑  Not easy/Somewhat Difficult ❑  Not easy at all/Very Difficult ❑ |
|  |  |  |  |
| 4. | How easy was it for you to hold the cup while feeding your baby? |  | Very Easy ❑  Somewhat Easy ❑  Neutral ❑  Not easy/Somewhat Difficult ❑  Not easy at all/Very Difficult ❑ |
|  |  |  |  |
| 5. | How much milk spilled while feeding your baby with this cup? |  | A lot ❑  Some ❑  Not much ❑  None ❑ |
|  |  |  |  |
| 6. | Is there anything you would like to tell us about this cup or anything else? |  | Yes [write in comments below] ❑  No ❑ |
|  |  |  |  |
|  | Comments:______________________________________________________________________________ | | |

**SECTION F. TRANSITIONS**

THE SECTION BELOW IS FILLED OUT BY THE RESEARCH ASSISTANT ONLY.

| 1. | what feeding assessment is this? | ❑1 GO TO Section G  ❑2 GO TO QUESTION 2  ❑3 GO TO SECTION G  ❑4 GO TO QUESTION 7 |
| --- | --- | --- |
|  |  |  |
|  | **FEEDING ASSESMENT NO. 2** |  |
| 2. | We will now give you the ____ cup to feed your baby with. | Medicine Cup ❑  _NIFTY CUP_ ❑ |
|  |  |  |
| 3. | RA COLLECTED FIRST CUP FROM THE PARTICIPANT. | YES ❑ |
|  |  |  |
| 4. | ra gave the other cup to the participant. | YES ❑ |
|  |  |  |
| 5. | ra has shown the video to the participant. | Yes ❑ |
|  |  |  |
| 6. | time ra gave the cup to the participant | _____:_____ Go to Section G.  (hour : minutes) |
|  |  |  |
|  | **FEEDING ASSESMENT NO. 4** |  |
| 7. | the ra gave cup 1 back to to the participant. | Yes ❑ |
|  |  |  |
| 8. | Go to Section G. AND THEN complete the in-hospital survey | Yes ❑ |

**SECTION G. FEEDING OBSERVATIONS**

|  | COMPLETE IMMEDIATELY AFTER THIS STUDY VISIT.  during the feeding you should observe how many times the caregiver or baby experienced each of the following below in **section b**. add them up at the end of the feed to note the total number of times this occurred and put them here. | | |
| --- | --- | --- | --- |
|  | **Feeding response monitoring:** |  |  |
| 1. | How many times did the baby cough? |  | \|  \|  \| \| --- \| --- \|   Total Number of times |
|  |  |  |  |
| 2. | How many times did the baby spit-up? |  | \|  \|  \| \| --- \| --- \|   Total Number of times |
|  |  |  |  |
| 3. | How many times did the baby dribble milk out of their mouth? |  | \|  \|  \| \| --- \| --- \|   Total Number of times |
|  |  |  |  |
| 4. | How many times was the feeding stopped because of uncoordinated breathing or swallowing?  [Provide guidance or obtain guidance from health care provider to improve this.] |  | \|  \|  \| \| --- \| --- \|   Total Number of times |
|  |  |  |  |
| 5. | How many times was the feeding stopped because the baby was lying down?  [guidance will be provided by RA or health care provider to improve this.] |  | \|  \|  \| \| --- \| --- \|   Total Number of times |
|  |  |  |  |
| 6. | How many times was the feeding stopped because the caregiver was pouring milk into the baby’s mouth?  [guidance will be provided by RA or health care provider to improve this.] |  | \|  \|  \| \| --- \| --- \|   Total Number of times |

|  | ❑FORM NOT COMPLETED. |  |  |
| --- | --- | --- | --- |
|  | WHY? |  |  |
