# Supplementary material for: A randomized crossover trial comparing the Nifty cup to a medicine cup in preterm infants who have difficulty breastfeeding at Komfo Anokye Teaching Hospital (KATH) in Kumasi, Ghana
Source: PLoS One. 2019 Oct 17;14(10):e0223951. doi: 10.1371/journal.pone.0223951 (PMC6797128; doi:10.1371/journal.pone.0223951)
Supplement: S2 Appendix — (DOCX) [file pone.0223951.s005.docx]

| In-Hospital Preference Survey **v4, 21April17**  **STUDY ID #______________ STAFF INITIALS:______________DATE** _____/_____/_______  (day/month/year) | | | |
| --- | --- | --- | --- |
|  | | | |
|  | Please answer the following questions regarding the feeding cups that you used. | | |
|  |  | | |
| 1. | Overall how much did you like using the Nifty Cup to feed your baby? |  | like a lot ❑  liked/ok ❑  neutral ❑  didn’t like ❑  really didn’t like ❑ |
|  |  |  |  |
| 2. | Overall how much did you like using the  Medicine Cup to feed your baby? |  | like a lot ❑  liked/ok ❑  neutral ❑  didn’t like ❑  really didn’t like ❑ |
|  |  |  |  |
| 3. | Overall, do you prefer the Nifty cup or the medicine cup to feed your baby? |  | Nifty Cup ❑  Medicine Cup ❑ |
|  |  |  |  |
|  | 3a. Why do you prefer this cup? |  |  |
|  |  |  |  |
|  |  |  |  |
| 4. | What other cups have you used to feed your baby? Do not include the Nifty cup or the medicine cup. |  | _No Other Cups Used_ ❑**→ (SKIP TO 6)**  Ghana Cup ❑  Bottle top ❑  _OTHER, SPECIFY:___________________________❑ |
|  |  |  |  |
| 5 | List the order of the cups according to your preference: |  | 1.____________________________________  2.____________________________________  3.____________________________________  4.____________________________________ |
| 6. | For the next questions, only think of the Nifty cup and the medicine cup. Which cup spilled less milk? |  | Nifty Cup ❑  Medicine Cup ❑  About the Same ❑ |
|  |  |  |  |
| 7. | Which cup is easier to use? |  | Nifty Cup ❑  Medicine Cup ❑  About the Same ❑ |
|  |  |  |  |
| 8. | Which cup was less likely to hurt the infant’s mouth? |  | Nifty Cup ❑  Medicine Cup ❑  About the Same ❑ |
|  |  |  |  |
| 9. | Which cup was made of material that you liked best? |  | Nifty Cup ❑  Medicine Cup ❑  About the Same ❑ |
|  |  |  |  |
| 10. | Which cup was easier to hold? |  | Nifty Cup ❑  Medicine Cup ❑  About the Same ❑ |
|  |  |  |  |
| 11. | Overall, how confident did you feel in being able to feed your baby with the Nifty cup?  [READ RESPONSES OUT LOUD] |  | Very confident ❑  Somewhat confident ❑  A little confident ❑  Not confident at all ❑ |
|  |  |  |  |
| 12. | How easy was it to use the tick markers on the Nifty feeding cup to measure the amount of milk you were giving your baby?  [READ RESPONSES OUT LOUD] |  | Very easy ❑  Somewhat easy ❑  Not easy or difficult ❑  Somewhat difficult ❑  Very difficult ❑  Did not use ❑ |

| 13. | Overall, how confident did you feel in being able to feed your baby with the medicine cup?  [READ RESPONSES OUT LOUD] |  | Very confident ❑  Somewhat confident ❑  A little confident ❑  Not confident at all ❑ |
| --- | --- | --- | --- |
|  |  |  |  |
| 14. | How easy was it to use the tick markers on the medicine cup to measure the amount of milk you were giving your baby?  [READ RESPONSES OUT LOUD] |  | Very easy ❑  Somewhat easy ❑  Not easy or difficult ❑  Somewhat difficult ❑  Very difficult ❑  Did not use ❑ |
|  |  |  |  |
| 15. | Did you hand express milk directly into the Nifty Cup? |  | Yes ❑  _No_ ❑**→ (SKIP TO 18)** |
|  |  |  |  |
| 16. | What did you like about hand expressing milk directly into the Nifty Cup? | | |
|  |  |  |  |
|  |  |  |  |
| 17. | What did you NOT like about hand expressing milk directly into the Nifty Cup? | | |
|  |  |  |  |
|  |  |  |  |
| 18. | Did you hand express milk directly into the medicine cup? |  | Yes ❑  _No_ ❑**→ (SKIP TO 21)** |
| 19. | What did you like about hand expressing milk directly into the medicine cup? | | |
|  |  |  |  |
|  |  |  |  |
| 20. | What did you NOT like about hand expressing milk directly into the medicine cup? | | |
|  |  |  |  |
|  |  |  |  |
| 21. | What do you use to capture breast milk from hand expression in the future? List all that you plan to use. |  | Nifty cup ❑  _MEDICINE CUP_ ❑  _OTHER, SPECIFY_ ❑ |
| 22. | What did you like about the Nifty Cup? |  |  |
|  |  |  |  |
|  |  |  |  |
| 23. | What did you NOT like about the Nifty Cup? |  |  |
|  |  |  |  |
|  |  |  |  |
| 24. | What could we do to improve the Nifty Cup? |  |  |
|  |  |  |  |
|  |  |  |  |
| 25. | What did you like about the medicine cup? |  |  |
|  |  |  |  |
| 26. | What did you NOT like about the medicine cup? |  |  |
|  |  |  |  |
|  |  |  |  |
| 27. | What could we do to improve the medicine cup? |  |  |
|  |  | | |

| 28. | GAVE THE PARTICIPANT DIAPERS |  | Yes ❑ |
| --- | --- | --- | --- |
|  |  |  |  |
| 29. | NOTIFIED ESTIMATE DATE OF PHONE CALL IN |  | Yes ❑ |
|  |  |  |  |
| 30. | THANKED PARTICPANT FOR PARTICIPATION |  | Yes ❑ |
|  |  |  |  |
| 31. | ANSWERED ANY FINAL QUESTIONS |  | Yes ❑ |
|  | NOTES | | |
|  |  |  |  |
|  |  |  |  |
| 32. | ❑FORM NOT COMPLETED. |  |  |
|  | WHY? |  |  |
