# Supplementary material for: A randomized crossover trial comparing the Nifty cup to a medicine cup in preterm infants who have difficulty breastfeeding at Komfo Anokye Teaching Hospital (KATH) in Kumasi, Ghana
Source: PLoS One. 2019 Oct 17;14(10):e0223951. doi: 10.1371/journal.pone.0223951 (PMC6797128; doi:10.1371/journal.pone.0223951)
Supplement: S3 Appendix — (DOCX) [file pone.0223951.s006.docx]

| Follow-Up Survey,  **v3, 16apr17**  **STUDY ID #______________ STAFF INITIALS:______________DATE** _____/_____/_______  (day/month/year) | | | | | |
| --- | --- | --- | --- | --- | --- |
| THIS SURVEY IS TO BE COMPLETED BY THE MOTHER OF THE INFANT 4 WEEKS AFTER HOSPITAL DISCHARGE.  Hi. This is [NAME]. I am part of the research team on the cup feeding study you participated in at KATH about one month ago. We mentioned during the in-hospital part of the study that we would call you a month after you left the hospital to see how you are doing. Is now a good time to talk? This will take about 5-10 minutes of your time. | | | | | |
|  |  | | | | |
|  | **SECTION A. INFANT HEALTH** | | |  |  |
|  |  | | |  |  |
| 1. | How is your infant doing? | | |  | check if Infant is Deceased ❑ |
|  |  | | |  |  |
|  | [IF INFANT IS DECEASED, TELL THEM YOU ARE SORRY AND END THE INTERVIEW] | | |  |  |
|  |  | | |  |  |
| 2. | How would you rate your infant’s health | | |  | Excellent ❑  Very Good ❑ Good ❑ Poor ❑  Very Poor ❑ |
|  |  | | |  |  |
|  | **SECTION B. BREAST MILK EXPRESSION** | | |  |  |
|  |  | | |  |  |
| 3. | What was your infant given to drink in the past 24 hours?  Anything else? [MARK ALL THAT APPLY] | | |  | Breast Milk ❑  Milk (other than breast milk) ❑ Plain Water ❑  Sugar or Glucose Water ❑ Gripe Water ❑  Sugar-Salt-Water Solution ❑ Fruit Juice ❑  Infant Formula ❑ Porridge ❑  Other: ________________________ ❑ |
|  |  | | |  |  |
| 4. | Are you currently expressing breast milk? | | |  | Yes ❑**→ (SKIP TO 4.A)**  No ❑**→ (SKIP TO 4.D)** |
|  |  | | |  |  |
|  | 4.A | IF YES: How many times have you expressed breastmilk in the past 24 hours? | |  | \|  \|  \| \| --- \| --- \| |
|  |  | | |  |  |
|  | 4.B | In the past 24 hours what method did you use to express your breastmilk? [CHECK ALL THAT APPLY] | |  | Hand expression ❑**→ (SKIP TO 5)**  Pump ❑  Other:___________________________ ❑ |
|  |  |  | |  |  |
|  | 4.C | What brand of pump did you use? | |  |  |
|  |  |  | |  | **→ (SKIP TO 5)** |
|  |  | | |  |  |
|  | 4.D | IF NO: How many weeks old was your baby when you stopped expressing breast milk? | |  | \|  \|  \| \| --- \| --- \| \| Weeks \| \| |
|  |  | | |  |  |
|  | **SECTION C. BREASTFEEDING** | | |  |  |
|  |  | | |  |  |
| 5. | Are you currently breastfeeding your baby? | | |  | Yes ❑  No ❑ Don’t know/Refused ❑ |
|  |  | | |  |  |
| 6. | How many times have you breastfed your baby in the past 24 hours? | | |  | \|  \|  \| \| --- \| --- \| |
|  |  | | |  |  |
| 7. | Have you exclusively breastfed your baby since you left the hospital? This means you have only given your baby your breast milk and no other food or milk. | | |  | Yes ❑  No ❑  Don’t know/Refused ❑ |
|  |  | | | | |
|  | **SECTION D. FEEDING TOOL USED MOST OF THE TIME** | | | | |
|  |  | | | | |
| 8. | Have you used any cup, bottle or other item to feed your baby since hospital discharge? [ASK AGAIN IF NO, JUST TO MAKE SURE] | | |  | Yes ❑  No ❑**→ (SKIP TO 18)**  Don’t know/Refused ❑**→ (SKIP TO 18)** |
|  |  | | |  |  |
| 9. | Which feeding cup, bottle or other item have you used **most of the time** since hospital discharge? | | |  | Nifty ❑  Medicine Cup ❑  GHANA Cup ❑  Bottle ❑  Other: __________________________ ❑ |
|  |  | | |  |  |
| 10. | Why did you use the [TOOL LISTED ABOVE] most of the time? MARK ALL THAT APPLY | | |  | Easy/Convenient ❑  Safe ❑  Less Spillage ❑  It was Free/I had it ❑  Not Stressful ❑  Soft/Liked the Material ❑  Other: __________________________ ❑ |
|  |  | | |  |  |
| 11. | Are you still using the [TOOL]? | | |  | Yes ❑**→ (SKIP TO 11.C)**  No ❑**→ (SKIP TO 11.A)** |
|  |  | | |  |  |
|  | 11.A | | If NO, how old was your baby the last time you used the [TOOL]? |  | \| \|  \|  \|  \|  \|  \| \| --- \| --- \| --- \| --- \| --- \| \| Weeks \| \| Days \| \| \| \| --- \| --- \| --- \| --- \| --- \| --- \| --- \| --- \| --- \| --- \|   Don’t know/Refused ❑ |
|  |  | | |  |  |
|  | 11.B | | When you used the [TOOL] the most, how many times per day did you use the [TOOL]? |  | \|  \|  \| \| --- \| --- \|     **(SKIP TO 12)** |
|  |  | | |  |  |
|  | 11.C | | If YES, how many times often have you use this [TOOL] in the past 24 hours? |  | \|  \|  \| \| --- \| --- \| |
|  |  | | |  |  |
| 12. | Overall how much did you like using the [TOOL] to feed your baby? | | |  | like a lot ❑  liked/ok ❑  neutral ❑  didn’t like ❑  really didn’t like ❑ |
|  |  | | |  |  |
|  | **SECTION E. OTHER TOOL USED TO FEED BABY** | | | | |
|  |  | | |  |  |
| 13. | What other cup, bottle or other item have you used to feed your baby since hospital discharge?  [IF THEY MENTION MORE THEN ONE, ASK:]  Which of these did you use the most?  USE THIS ANSWER AND ONLY SELECT ONE TOOL. | | |  | Nifty ❑  Medicine Cup ❑  GHANA Cup ❑  Bottle ❑  I have not used Any Other ❑**→ (SKIP TO 18)**  I have used all equally ❑  Other: __________________________ ❑ |
|  |  | | |  |  |
| 14. | Why did you use this [TOOL]? | | |  | Easy/Convenient ❑  Safe ❑  Less Spillage ❑  It was Free/I had it ❑  Not Stressful ❑  Soft/Liked the Material ❑  Other Tool Not Clean ❑  Other: __________________________ ❑ |
|  |  | | |  |  |
| 15. | Are you still using the [TOOL]? | | |  | Yes ❑**→ (SKIP TO 15.C)**  No ❑**→ (SKIP TO 15.A)** |
|  |  | | |  |  |
|  | 15.A | | If NO, how old was your baby the last time you used the [TOOL]? |  | \| \|  \|  \|  \|  \|  \| \| --- \| --- \| --- \| --- \| --- \| \| Weeks \| \| Days \| \| \| \| --- \| --- \| --- \| --- \| --- \| --- \| --- \| --- \| --- \| --- \|   Don’t know/Refused ❑ |
|  |  | | |  |  |
|  | 15.B | | When you used the [TOOL] the most, how many times per day did you use the [TOOL]? |  | \|  \|  \| \| --- \| --- \|     **(SKIP TO 16)** |
|  |  | | |  |  |
|  | 15.C | | If YES, how many times often have you use this tool in the past 24 hours? |  | \|  \|  \| \| --- \| --- \| |
|  |  | | |  |  |
| 16. | Overall how much did you like using the [TOOL] to feed your baby? | | |  | like a lot ❑  liked/ok ❑  neutral ❑  didn’t like ❑  really didn’t like ❑ |
|  |  | | |  |  |
|  | **SECTION F. OTHER TOOL USED TO FEED BABY** | | |  |  |
|  |  | | |  |  |
| 17. | What other cup, bottle or other item have you used to feed your baby since hospital discharge?  [IF THEY MENTION MORE THEN ONE, ASK:]  Which of these did you use the most?  IF THEY HAVE USED ANOTHER TOOL, THEN ASK SECTION D QUESTIONS USING ANOTHER PAGE. | | |  | Nifty ❑  Medicine Cup ❑  GHANA Cup ❑  Bottle ❑  I have not used Any ❑  Other: __________________________ ❑ |
|  |  | | |  |  |
| 18. | Is there anything you would like to tell us about this cup or anything else? | | |  | Yes ❑  No ❑ |
|  |  | | |  |  |

Comments:

_____________________________________________________________________________________________________

|  | ❑FORM NOT COMPLETED. |  | WHY? |
| --- | --- | --- | --- |
|  |  |  |  |
