# Supplementary material for: A randomized crossover trial comparing the Nifty cup to a medicine cup in preterm infants who have difficulty breastfeeding at Komfo Anokye Teaching Hospital (KATH) in Kumasi, Ghana
Source: PLoS One. 2019 Oct 17;14(10):e0223951. doi: 10.1371/journal.pone.0223951 (PMC6797128; doi:10.1371/journal.pone.0223951)

###

# A Randomized Crossover Trial of the Nifty Feeding Cup and a

# Medicine cup in preterm infants who have difficulty breastfeeding

# (‘The Feeding Cup Study’)

Study PI: Christy M. McKinney, Ph.D., MPH

Co-PI: Patricia S. Coffey, Ph.D., MPH

Site PI: Gyikua Plange-Rhule, MBChB

Version: 7

January 24, 2019

# Contents

A Randomized Crossover Trial of the Nifty Feeding Cup and a 1

Medicine cup in preterm infants who have difficulty breastfeeding 1

(‘The Feeding Cup Study’) 1

Contents 2

List of Abbreviations 4

1.0 Background and rationale for the study 5

1.1 The Nifty Feeding Cup Premise 5

1.2 Background and Context 5

1.3 Justification 6

1.4 Significance 7

2.0 Study objectives 7

2.1 Goal and Objective 7

2.2 Aim and Hypotheses 7

2.3 Statistical Justification 7

3.0 Study design 8

3.1 Statistical Analysis 8

4.0 Research participants 8

4.1 Study Population 8

4.2 Study Eligibility: Inclusion and Exclusion Criteria Inclusion 9

5.0 Study procedures 10

5.1 Visits 11

5.1.1 Visit 1. Baseline (Day 1, Hour 0) 11

5.1.2 Visit 2. Cup 1 Feed 1 (Day 1, >4 hours after V1) 11

5.1.3 Visit 3. Cup 1 Feed 2 (Day 1, >2 hours after V2) 11

5.1.4 Visit 4. Cup 2 Feed 1 (Day 2, >4 hours after V3) 11

5.1.5 Visit 5. Cup 2 Feed 2 (Day 2, >2 hours after V4) 11

5.1.6 Discharge Data Abstraction 12

5.1.7 Visit 6. Follow-up Survey (Week 4 post-discharge) 12

5.2 Procedures and Evaluations 12

5.2.1 Intervention 12

5.2.2 Outcomes 12

5.2.3 Case Report Forms 13

5.2.4 Other Measures 13

5.2.5 Data Abstraction 14

5.3 Retention 14

5.4 Withdrawal 14

6.0 Recruitment 14

7.0 Consent process 15

8.0 Study product(s) 16

9.0 Risks 17

10.0 Mitigation of risks 18

11.0 Benefits 18

12.0 Study and safety monitoring 18

13.0 Managing and reporting adverse events 19

14.0 Managing and reporting unanticipated problems or protocol deviations 19

15.0 Confidentiality and data management 20

15.1 Quality Control and Quality Assurance 20

15.1.1 Quality Assurance (QA) 20

15.1.2 Quality Control (QC) and Auditing 20

15.1.3 Staff Training 20

15.2 Data Handling and Record Keeping 21

15.2.1 Overview 21

15.2.2 Consent Documentation and Translation 21

15.2.3 Data Corrections 21

15.2.4 Data Management Responsibilities 21

15.2.5 Data Capture Methods 21

15.2.6 Study Records Retention 21

15.3 Participant Confidentiality and Data Storage 21

15.3.1 Participant Confidentiality 21

15.3.2 Data Storage 22

16.0 Study costs 22

17.0 Care for injury 22

18.0 Compensation 22

19.0 Investigator responsibilities 22

Appendices: 22

# List of Abbreviations

| AE | Adverse Event |
| --- | --- |
| CRF | Case report form |
| GEE | Generalized Estimating Equations |
| IRB | Institutional Review Board |
| KATH | Komfo Anokye Teaching Hospital |
| MOP  N | Manual of Procedures  Number (typically refers to number of participants) |
| NGO | Non-governmental Organization |
| PATH | PATH |
| PI | Principal Investigator |
| QA | Quality Assurance |
| QC | Quality Control |
| RA  REDCap | Research Assistant  Research Electronic Data Capture |
| SAE | Serious Adverse Event |
| SCH | Seattle Children’s Hospital |
| SCRI | Seattle Children’s Research Institute |
| UNICEF | United Nation’s Children’s Fund |
| UP | Unanticipated Problem |
| UW | University of Washington |
| V1, V2….V6 | Visit 1, Visit 2 and so on |
| WHO | World Health Organization |

# 1.0 Background and rationale for the study

We will establish an evidence base for the Nifty Feeding Cup by evaluating its effectiveness and caregiver satisfaction. We will conduct a randomized crossover trial that compares the Nifty Feeding Cup to a standardized, generic medicine cup used to feed preterm infants with breastfeeding difficulties at Komfo Anokye Teaching Hospital (KATH) in Kumasi, Ghana. This work has the potential to improve the health and survival of over 9 million infants born in South Asia and Africa.

1.1 The Nifty Feeding Cup Premise

The Nifty Feeding Cup (Figure 1) is a simple, safe, easy-to-clean, ergonomic, and affordable tool designed to optimize feeding and the efficient hand expression of breast milk to newborn preterm infants and other infants with breastfeeding difficulties. Its unique shape minimizes spillage, enables the infant to participate in the feed, is large enough for direct hand expression of breast milk, and its soft material minimizes injury to the infant’s mouth and helps control flow. The Nifty Feeding Cup offers a standard cup for cup feeding infants in low resource settings where none currently exists.


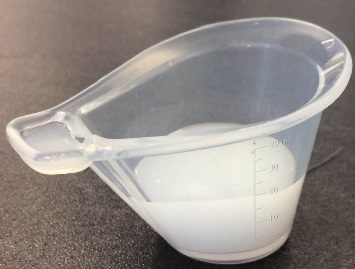


Figure 1. Nifty Feeding Cup by Laerdal

1.2 Background and Context

Feeding devices used in high-resource settings for infants with breastfeeding difficulties such as nasogastric tubes, bottles, and breast pumps are impractical and unhygienic in settings that lack clean water and electricity.^1^ The World Health Organization (WHO) and United Nations Children’s Fund (UNICEF) recommend hand expression of breast milk and the use of a small cup to feed newborns with breastfeeding difficulties in low-resource settings.^2-4^

No standard feeding cup for these infants exists. Non-specific cups such as small shot glasses, medicine cups, the paladai (a 10-ml beaked cup used in south India), and generic drinking cups are used worldwide. Other tools used include spoons and syringes. Generic cups have several key disadvantages. Most cups have relatively wide rims that make it difficult for the mother to control the rate at which milk flows into a newborn’s mouth and makes it difficult to maintain a constant bolus of milk to the infant at all times. Poor milk access and flow control decreases intake and lengthens feeding times which decreases net weight gain because of the excess energy it takes to feed. The risk of infection is higher because cups are often made of metal or hard plastic which often cuts the thin, tender skin surrounding the preterm newborn’s mouth. Conventional cups are too small for hand expression of breast milk, which means breast milk is transferred from other, possibly contaminated containers prior to feeding to the newborn.

Cups are more easily sanitized than bottles and only require the infant be able to swallow and breathe, which demands less energy and skill than the more complex suck/swallow/breathe mechanism needed for breastfeeding.^5^ Preterm infants developing their suck-swallow-breathe mechanism often require supplemental feedings until they can exclusively breastfeed.^6^ In low-resource settings, an optimized cup is the best way to provide these supplement feeds. Appropriate cup feeding of a newborn is achieved by delivering milk to the edge of the infant’s mouth and enabling the infant to lap or sip the milk at her own pace in a calm manner that encourages rapid, optimal intake and promotes mother-infant bonding.

The wide rims on generic cups make it difficult for the inexperienced cup feeding and new mother to control the rate of milk flow to a newborn’s mouth. Rapid or inconsistent rate of milk flow from wide-rimmed cups is an incorrect feeding pattern.^7^ This method results in spillage, stresses the infant, and often causes the infant to cough or aspirate which in turn reduces essential nutritional intake, increases feeding duration and fatigue, and frustrates the mother whose milk is laboriously hand expressed to only then be spilled and wasted. Over a short period of time, this type of feeding may quickly result in chronic insufficient caloric intake that reduces endurance and increases a vulnerable neonate’s risk of undernutrition, failure to grow, infection, and death. For example, the difference between adequate and inadequate intake can be as little as 2 teaspoons per feed for a 33-week preterm infant weighing 1,800 grams.

The Nifty Feeding Cup (Figure 1) addresses this problem by use of the reservoir which makes a bolus of milk constantly available to the infant at all times and minimizes spillage. Similar to breastfeeding, this allows the infant to participate in the feed by controlling how much milk is taken in and allows them to coordinate their suck, swallow, breathing during the feeding cycle. Minimizing spillage preserves precious hand expressed breast milk.

The original NIFTY Cup was designed by a team of investigators from Seattle Children’s Hospital, PATH and the University of Washington. Since 2015, we have also collaborated with Laerdal Global Health, our industrial partner, on the design. We conducted formative research on the Nifty Feeding Cup design in neonatal intensive care units in hospitals in India and Tanzania. An iterative design process was employed incorporating feedback from testing to improve the design. The cup was well received. In some design tests we compared the NIFTY™ to the paladai, a small beaked cup used widely in South India. Mothers and lactation providers preferred the NIFTY Cup to the paladai because of: 1) less spillage, 2) fewer cuts to the mouth, and 3) its larger size. Because we have not widely tested the NIFTY Cup in Africa and because there is no uniformity in cups used, we will conduct the Nifty Feeding Cup clinical trial with partners in Ghana.

In sum, we propose the Nifty Feeding Cup will improve the feeding experience of the mother-infant pair and will become the standard cup for feeding infants with breastfeeding difficulties in low resource settings.

1.3 Justification

Generic cups are subpar at feeding infants with breastfeeding difficulties in low resource settings. We expect the Nifty Feeding Cup to succeed because it is specifically designed to optimize intake, reduce feeding spillage, duration, and infection risk. We posit it will decrease cup feeding problems, and improve the caregiver’s experience of cup feeding her infant. A better cup for feeding infants with breastfeeding difficulties is needed. The Nifty Feeding Cup will fill this need.

1.4 Significance

Little attention has been given to feeding newborns with breastfeeding difficulties yet the potential impact is huge. There are over 9 million preterm infants and other infants with potential breastfeeding difficulties (e.g. craniofacial anomalies, orphans) born each year in Africa and South Asia. These infants are at high risk of death and malnutrition. Safe, early, frequent, and sufficient nutrition is an essential component of helping these babies grow, develop and survive. This will be the first comparative study of the Nifty Feeding Cup and the first research study of the Nifty Feeding Cup in Africa. Given the large numbers and the ramifications of poor early feeding, this study has the potential for global impact.

# 2.0 Study objectives

2.1 Goal and Objective

The goal is to establish an evidence base for the Nifty Feeding Cup and to evaluate its effectiveness and patient caregiver satisfaction. Our objective is to conduct a randomized crossover trial that compares the Nifty Feeding Cup to a standardized, generic cup used to feed preterm infants with breastfeeding difficulties at KATH in Kumasi, Ghana.

2.2 Aim and Hypotheses

We aim to compare the Nifty Feeding Cup to a standardized, generic cup in up to 200 caregivers and their preterm infants. We will test the hypotheses that Nifty Feeding Cup feeding compared to generic cup feeding will result in:

Primary:

1. Less spillage.
2. Greater caregiver satisfaction

Secondary:

1. Greater intake of milk (accounting for spillage, exploratory)
2. Shorter feeds (exploratory)
3. Greater caregiver satisfaction 4 weeks post-discharge

Our aim and hypotheses were selected based on the most informative outcomes given the scope of the funding. The above hypotheses are framed in terms of the statistical alternative which is typically more intuitive to non-statisticians.

2.3 Statistical Justification

Power for this study was determined by our primary hypotheses for the study H_A_ 1 related to amount of spillage and H_A_ 2 related to intake of milk. With 150 (up to 200) pairs, we estimate we will be able to detect at least a 5% difference in spillage or intake with 90% power and an alpha of 0.05 if no design effects are present. Carryover effects occur when a participant learns from cup feeding with the first cup which then affects the outcomes when feeding with the second cup. We will be able to statistically evaluate if carryover effects are present. If carryover effects are observed, we will have 80% power to detect an average 5% difference in spillage or intake. These estimates are based on studies report differences in intake and spillage.^8-10^ We used the ‘power’ command in Stata 13.0 to estimate power using a two-sided paired t-test. We selected a conservative difference based on other studies, which showed differences between 6.3 and 7.8ml.^8-10^ We used a published estimate of a cup feeding standard deviation of 11^10^ and conservative correlations ranging from 0.02 to 0.5. This power calculation is also applicable to a 5% difference caregiver satisfaction between the two cups.

# 3.0 Study design

We will conduct a randomized crossover trial in mothers/caregivers with a preterm infant in the mother-baby unit at Komfo Anokye Teaching Hospital (KATH) in Kumasi, Ghana. Each caregiver/infant pair will use the Nifty Feeding Cup for about 24 to 36 hours and the standardized generic cup for about 24 to 36 hours. Cross-over means each caregiver/infant pair will use both cups, with the order of the cup used first and second randomized. See Section 8 for further description of study products.

3.1 Statistical Analysis

The data analyst will be blinded to the cup assignment We will use generalized estimating equations for a crossover design to assess intake, spillage, and feeding duration and a sign test to assess the caregiver’s preference. Covariates may include factors such as amount of prior feeding, type of nutrition, number of prior cup feeds with assigned cup, and number of cup feeds between measured feedings. Most covariates typically accounted for (maternal age, other demographics) will not be included because the comparison is the same caregiver/infant pair.

We will conduct exploratory analyses stratifying on age (0-14 days and 15-28 days) and gestational age (<34 weeks and 34-36 weeks) to evaluate potential effect modification by these factors. These are exploratory since this study was not powered to detect effect modification. For each cup separately, we will compare estimates for those assigned the cup first to estimates for those assigned the cup second to evaluate for design effects. If design effects are observed we will analyze our data comparing those first assigned the Nifty cup to those first assigned to the medicine cup, dropping the data from the second cup and removing the cross over design from our study. We will use Stata 13.0 for all statistical analysis. Consistent with our power calculations, a two-sided p-value of 0.05 will be used.

A strength of our statistical approach is that participants with outcome data for at least one feeding assessment are able to be included in our GEE analyses even if other time points are missing. We will evaluate the potential effect of other missing data on our associations using multiple imputation methods.^12^

# 4.0 Research participants

4.1 Study Population

We plan to enroll up to 200 caregiver and their infant while the pair is in the mother-baby unit at KATH. The following criteria outline inclusion and exclusion criteria for being in the study.

Based on numbers from KATH, in 2013 there were 567 infants born with birth weights below 1800 grams (gestational age is not available) who live at least 28 days. Given most term infants are greater than 1800 grams at birth, we presume this is our recruitment pool. Based on these numbers, we estimate we will need to recruit 26% (150/567) of the clinical population over the course of a year.

4.2 Study Eligibility: Inclusion and Exclusion Criteria Inclusion

In order to be eligible to participate in this study, the caregiver-infant pair must meet all of the following. The information in [brackets] specifies where this information will typically be ascertained. A caregiver is the person primarily responsible for feeding the infant. This is typically the mother. In cases where the mother is unwell or deceased, we may enroll a caregiver who is a biological family member designated as the person primarily responsible for feeding the infant. Relations who meet this criterion include a biological grandmother or aunt of the infant. The admitting doctors will do the scoring using the physical characteristics of the modified Dubowitz assessment tool (see final page of this document). The admitting doctors will be House Officers supervised by Paediatric Residents and will be qualified (BSc Human Biology, MB ChB – which is analogous to MD) and members of the Ghana College or the West Africa College of Physicians.

**Inclusion**

- Infant
  - Infant was born preterm (<37 weeks gestational age) at time of birth [chart]
  - Infant’s corrected gestational age is <37 weeks per the modified Dubowitz score on date of enrollment [chart].
  - Infant diagnosed with feeding difficulties [provider]
  - Infant is a patient in the mother-baby unit at KATH [chart/location]
  - Infant is clinically indicated to start cup feeding (including an infant who has a nasogastric tube and is cup feeding or indicated to start cup feeding) [provider]
  - Infant has an anticipated hospital stay that is at least 48 hours [provider]
- Caregiver:
  - Caregiver must be at least 18 years of age [caregiver]
  - Caregiver must be one of the following biologic family members of the infant [caregiver]:
    - Mother
    - Grandmother
    - Aunt
  - Caregiver must self-identify as the primary feeder of the infant [caregiver]
  - Prior experience feeding the potential infant participant with nipple feeding (e.g. breast feeding, bottle feeding, or nasogastric [NG] tube) is okay but is not required [caregiver].
  - Caregiver verifies willingness to comply with all study procedures [caregiver]

**Exclusion**

- Infants with a congenital anomaly except for minor anomalies (e.g. an extra digit or ear tag is okay) [provider]
- Infant has other condition or situation that makes them unlikely to be able to comply with study procedures. Examples include the infant anticipated to not be in hospital long enough, infant has a suspected intestinal obstruction, or necrotizing enterocolitis. [provider]
- Infants without a mother, grandmother, or aunt caregiver [provider or caregiver]
- Infant has been enrolled in another study at KATH that would interfere with his/her ability to participate in this study.

# 5.0 Study procedures

Study participants will be asked to cup feed their infant with the generic cup and the Nifty Feeding Cup in the randomized order assigned. The caregiver will feed their infant with each cup at least 2 times before we measure feedings with the cup. Mothers will not be asked to exclusively cup feed during this study. Preterm infants started on cup feeding will typically start with a cup and then gradually transition to breastfeeding. Mothers are counseled to attempt breast feeding while their infant is using the feeding cup. Study procedures are aligned with KATH practice so the mother will not do anything different than what is clinically indicated as best for mother and baby.

Since infant participants are inpatients, the term ‘visit’ is meant loosely to indicate the sequential study activities that will take place. Research staff may check-in with the caregiver at any time during the participation in the study to coordinate study activities. The day and time of each study visit is an estimate but each visit will be completed before the next. To ensure the infant is clinically indicated to cup feed, prior to each feed we measure, the research assistant (RA) will consult with a provider to make sure the infant can be cup fed.

The caregiver will feed their infants the type of milk/formula in amounts determined by the clinical provider. The study team will not be involved in providing guidance regarding type of milk or amount recommended other than tracking it.

If the infant and/or caregiver have health, social or other problems that arise in the course of study participation that prevent either of them from participating in study visits, we will discontinue them from the study

Details of the tools (e.g. Surveys) and procedures (e.g. Feeding Assessment) used at each visit are outlined in Section 6 below. Also refer to the **Schematic of Study Procedures** found immediately after the Protocol Summary and the **Visit Activities Schedule**, an Appendix to the protocol.

We estimate the RAs will each be able to manage a workload of 2-3 participants per week and that based on this rate, it will take about 1 year to complete enrollment and data collection activities.

Study participants will be discharged from hospital when clinically appropriate. Participants will not be retained in hospital to finish the study procedures.

5.1 Visits

### 5.1.1 Visit 1. Baseline (Day 1, Hour 0)

After the consent process, the RA will conduct the Baseline visit. She will:

- Obtain the random assignment of which cup to introduce first
- Administer the Baseline Survey
- Measure the infant’s current weight and length.
- Provide the caregiver with assigned Cup 1 and the instructions for use.

### 5.1.2 Visit 2. Cup 1 Feed 1 (Day 1, >4 hours after V1)

To allow the caregiver time to feed her infant with Cup 1 for at least two times without being observed by the RA, we will conduct this visit >4 hours after V1. The RA will periodically check in to see if at least two feedings have occurredand then conduct V2. During Visit 2, the RA will:

- Conduct the Feeding Assessment

### 5.1.3 Visit 3. Cup 1 Feed 2 (Day 1, >2 hours after V2)

The RA will measure a second feeding at V3. There may or may not be feedings between V2 and V3. During Visit 3, the RA will:

- Conduct the Feeding Assessment
- Remove Cup 1 so it is not inadvertently or preferentially used over Cup 2
- Provide the caregiver with Cup 2 and the instructions for use.

### 5.1.4 Visit 4. Cup 2 Feed 1 (Day 2, >4 hours after V3)

To allow the caregiver time to feed her infant with the Cup 2 for at least two times without being observed by the RA, we will conduct this visit >4 hours after V3. The RA will periodically check in to see if at least two feedings have occurred and then conduct V4. During Visit 4, the RA will:

- Conduct the Feeding Assessment

### 5.1.5 Visit 5. Cup 2 Feed 2 (Day 2, >2 hours after V4)

This is the last visit while the participant is an inpatient in the hospital. The RA will measure a second feeding at V5. There may or not be feedings in-between V4 and V5. We will provide the thank you at this visit. During Visit 3, the RA will:

- Conduct the Feeding Assessment
- Conduct In-Hospital Preference Survey

Return Cup 1 to the caregiver, Cup 2 will remain with the caregiver after completion of the study procedures.

- Provide thank you
- Schedule Follow-Up Survey

### 5.1.6 Discharge Data Abstraction

We will schedule the Follow-up Survey at the end of Visit 5 based on anticipated discharge date. We will subsequently abstract actual discharge date and health information from the record (See Section 5.2.5 below). We may reschedule visit 6 if the discharge date is substantially different than anticipated.

### 5.1.7 Visit 6. Follow-up Survey (Week 4 post-discharge)

During Visit 6 the RA will conduct the Follow-up Survey by telephone or in person for those who return to clinic for their 1 month follow-up and thank the participant verbally one last time for their participation.

5.2 Procedures and Evaluations

### 5.2.1 Intervention

Random Assignment. Random assignment will be given using sealed numbered sequential envelopes prepared by the PI and stored at KATH. PI McKinney, who will do the statistical analysis, will not prepare the randomization schedule we will use. PI Coffey and colleagues at PATH will prepare the randomization schedule.

Cup. We will provide all cups used for the study. The first cup used as determined by the random assignment is called Cup 1. The other cup is called Cup 2. We will use the Nifty Feeding Cup being produced by Laerdal Global Health. We will use a small generic medicine cup as the comparison.

Instructions for Use. We will show a short video that shows how to use the Nifty Feeding Cup and a short video that shows how to use the generic cup. Videos will be shown to participant on a table or laptop device. If the participant has a video capable phone, the video may be loaded on to their personal device for reference. The video will include instructions on how to use and clean the cups being used. These videos have been provided to the IRB.

### 5.2.2 Outcomes

Outcomes related to intake, spillage, and duration will be measured at each Feeding Assessment, which will take place at Visit 2 through 5. Caregiver satisfaction will be measured during Feeding Assessment related to the experience of that feed (V2-V5). Caregiver satisfaction will be measured with the In-Hospital Preference Survey (V5) and the Follow-up Survey (V6).

Milk Intake. We will measure amount (in ml) and weight (in grams) of milk in the cup before the feeding. We will measure the amount and weight of milk in the cup after the feeding. If the cup is refilled, we will measure the amount and weight before and after the refill. The amount and weight will be recorded on the Feeding Assessment case report form. We will use a digital scale to determine all weight measurements. The weight per ml will be estimated based on the baseline measure and used to adjust the gross measure of intake by subtracting out spillage that occurred during the feed. We will calculate intake per feed from these measures less the amount spilled.

Spillage. We will account for spillage of milk by using a baby bib cloth for all feedings. Each caregiver-infant pair will be provided with a bib cloth for each observed feeding. The bib cloth will be weighed before and after each feed and the weights recorded on the Feeding Assessment form. The difference between the pre and post weights will be used as the measure of the amount spilled. A digital scale will be used and milk weight will be in grams. Spillage will be reported as a percent with the amount of milk in grams spilled/mopped up divided by the total amount of milk weighed in grams less the total amount of milk not used.

Caregiver Satisfaction. Our primary measure of caregiver’s satisfaction will be the cup she prefers [secondary] and rated satisfaction [primary] which will be recorded in the In-Hospital Preference Survey completed after the caregiver has finished the feeding portion of the study (V5). A secondary measure will be what the caregiver reports uses most of the time measured in the Follow-up Survey 4 weeks post-discharge (V6). We will also measure the caregiver’s satisfaction with each cup after each feeding measured in the Feeding Assessment and in the In-Hospital Preference Survey (V2-V5).

Duration of Feed. The RA will measure duration of feeding in minutes and seconds with a digital timer. The timer will begin at the time the cup first touches the infant’s lip and stop when the cup last leaves the infant’s mouth. If there are stops and starts in the feeding (e.g. mother needs to express more milk), we will also record this information. The time measured will be recorded on the Feeding Assessment form.

### 5.2.3 Case Report Forms

Case report forms will record intervention and outcome data as well as data on other covariates.

Baseline Survey, V1. The baseline intake will record cup assignment, infant weight, length, demographics and infant and maternal feeding and health characteristics (e.g. co-morbidities, type of delivery, etc). It is estimated to take about 10-15 minutes to complete.

Feeding Assessment, V2-V5. This case report form will be used to record outcome information collected on milk intake, spillage and duration of feed and the cup used described below and caregiver’s immediate experience using the cup. It will also collect information about prior feedings and time since last feed. Each Feeding Assessment will last as long as the feeding plus about 5 minutes.

In-Hospital Preference Survey, V5. This survey will collect information on the caregiver’s experience using both cup and her preferences. It will take about 5 minutes to complete.

Follow-up Survey, V6. This survey will assess cup feeding practices, infant health, and maternal satisfaction with the cup. It will take about 10 minutes to complete.

### 5.2.4 Other Measures

Infant Weight and Length, V1. We will weigh and measure the length of the infant using standard equipment used in the mother-baby unit. We will follow WHO guidelines on measuring weight and height.^11^ These procedures will take about 5-10 minutes.

### 5.2.5 Data Abstraction

Prescreening Log. We will abstract participant information (e.g. name, bed, cup feeding status, etc.) onto the prescreening log in order to track eligibility of potential participants.

Eligibility Checklist. We will abstract and record information about eligibility on this form. Information on the eligibility screener will come from the clinical records, the provider and the caregiver. All eligibility information will be collected prior to determining eligibility and enrolling a participant.

For assessing eligibility related to congenital anomalies, we will refer to a standardized list of minor congenital anomalies.^13^ If the infant has a congenital anomaly, the RA will check the list to determine if it is minor.

Chart Review of Discharge. We will record the date of discharge on a discharge log to determine how long the caregiver has been cup feeding at the time of the Follow-up Survey. To the extent available, we will collect age, weight, and feeding status (current breastfeeding status, cup feeding prescribed and volumes, supplemental feeding if required).

5.3 Retention

Because our study is predominantly conducted in inpatients and is short, we anticipate we will have minimal loss to follow-up.

5.4 Withdrawal

Subjects are free to withdraw from participation in the study at any time upon request. The study team may withdraw subjects who are not compliant with study visits or data collection procedures or who meet an exclusion criterion after enrollment. We may also terminate a study subject’s participation in the study if any medical condition, event or situation occurs such that continued participation in the study would not be in the best interest of the subject. If a participant decides to withdraw from the study, we will honor that decision, but will also do our best to understand why the caregiver/parent wishes to withdraw and respond to their concerns. We will document the reason for study withdrawal on a document that tracks study participation that we will maintain in Box.

If we discover a previously unrecognized, exclusionary condition that existed prior to the subject’s enrollment in our research, we will not conduct the remaining study procedures and explain that because they are not eligible we are discontinuing their participation. Data generated by ineligible subjects (previously unrecognized) will not be used in our analyses. The caregiver/parent will still be given the Nifty and generic feeding cups, five diapers and the bib cloth used to weigh spillage as a thank you for their time and effort.

# 6.0 Recruitment

The purpose of pre-screening is to identify potentially eligible participants and those who will soon become eligible in order to identify who to approach about the study. Prescreening will consist of (1) reviewing and abstracting clinical rosters and medical records onto a prescreening log to identify and track potentially eligible participants and (2) consulting with clinical providers who can inform on eligibility criteria. The study RA in consultation with the site PI are responsible for reviewing clinical rosters and medical records and consulting with clinical providers on potentially eligible participants.

When feasible, a provider will introduce the study to the caregiver/parent to see if they are interested in learning more using a standardized recruitment script. Because of their volume of patients, it may not be practicable for a provider to always introduce the study. On these occasions, the RA will approach the potential participant after establishing eligibility via records and via consultation with the provider. The RA will approach the caregiver/parent and provide a brief overview of the study using the recruitment script noted above. If the caregiver/parent is interested, the RA will assess eligibility criteria. For those interested and eligible, the RA will proceed with consent.

Some caregiver-infant pairs approached may not be eligible at the time screened, but may become eligible in the future (e.g. infant not yet indicated for cup feeding). We will seek verbal permission to re-approach those interested who are not eligible but maybe come eligible. Those who indicate they are not interested will not be re-approached.

# 7.0 Consent process

Informed consent is a process that is initiated prior to the individual agreeing to participate in the study and continues throughout study participation. Discussion of risks and possible benefits of study participation will be conducted with the caregiver. A consent form describing the study procedures and risks will be given to the caregiver. The potential risks and benefits and study activities will also be provided to caregivers orally. To ensure privacy for the consent process, we use a nurse manager room to conduct the consent process. Alternatively, we will use a screen to increase privacy on the ward if the room is not available for some reason.

We will obtain consent from the caregiver (typically the mother).  Although the mother may not be able to participate it the study, she may be able to consent for the infant and identify a relative caregiver. In cases where the mother is unavailable, we will seek consent from the father of the infant and he will identify a relative caregiver. We will then seek consent for the relative caregiver (an aunt or grandmother).  To accommodate this, we would like to revise the consent form to include two statements at the end that the person can tick as noted:

I am the parent of the child.

I am the caregiver of the child.

In cases where the mother (both parent and caregiver) is unavailable, we will use two forms for one infant to show consent of the parent (father) and consent of caregiver (aunt or grandmother).

After reading the consent in English to the potential participant, the person consenting will also explain in Twi if desired. The RAs will be bilingual and able to provide accurate Twi translation. Consent forms will be reviewed with the caregiver/parent orally. The research staff will explain the research study to the caregiver/parent and answer any questions that may arise. The caregiver/parent will sign the informed consent document prior to any study-related assessments or procedures. Caregivers/parents will be given the opportunity to think about it prior to agreeing to participate.

A copy or duplicate of the signed informed consent document will be given to the caregiver/parent for their records. A copy is a photocopy of the original signature consent. A duplicate is a second consent form with all originally signatures. A duplicate will be used when a copier is not readily available. The rights and welfare of the caregiver/parent will be protected by emphasizing to them that the quality of the infant’s clinical care will not be adversely affected if they decline to participate in this study. The caregiver/parent may withdraw consent at any time throughout the course of the study.

Informed consent will take place *at least* 24 hours after the birth of the child to allow for birth related care to take place, including the identification of the infant as preterm. The RA will obtain permission from the health care provider to approach the caregiver/parent about the study as part of the screening process. Those participants unable to write their name have the option of providing a fingerprint and having the RA write in their name. Fingerprint consent will be observed by a witness who will also sign the consent form. The consent witness will be an impartial witness and not a family member. We have identified several types of individuals that could serve as impartial consent witnesses for mothers who are not literate. These include the following: (1) hospital administrators, (2) technology support individuals, (3) nurses on other surgical or medical wards (those in the mother-baby unit will not serve as consent witnesses), or (4) records officers.

# 8.0 Study product(s)

The Nifty Feeding Cup by Laerdal will be the version of the NIFTY cup used for this study. The Nifty Cup manufactured by Laerdal Global Health (see: <http://www.laerdalglobalhealth.com/doc/2576/Nifty-Feeding-Cup#/Info> ) is a reusable product for feeding breast milk to newborns with breastfeeding difficulties (Figure 1). It is a simple to use, easy to clean and culturally appropriate feeding solution which allows the infant to control the pace of feeding. Product specifications are as follow:

- Reusable for use by multiple newborns
- Suitable for reprocessing with the following methods:
  - Steam autoclaving at 136 °C, 10-20 minutes
  - Chemical disinfection with 0.5% chlorine solution, 20 minutes, and rinsed 3 times with clean water afterwards
  - Boiling in water, 10 minutes
- Made with silicone rubber
- Safe for food contact according to *USFDA 21 CFR 177.2600 regulation* Mass: Approx. 23.5 g
- Volume: 5 to 40 ml; the cup has tick marks every 5ml and numerals every 10 ml
- Operating Temperature: -18 °C to +50 °C
- Storage temperature: -40 °C to +60 °C
- Not made with natural rubber latex

For every batch of silicone materials used in the Nifty feeding cup by Laerdal, material samples are sent to an independent test lab for testing to USFDA requirements applicable for food contacting rubber materials, 21 CFR 177.2600.

In all geographies, the Nifty Cup is a being commercialized by the manufacturer as a cup for general infant feeding and not a medical device. To implement this decision, Laerdal Global Health:

o   Will only label/promote it for general infant feeding—there will be no references to specific health conditions such as cleft palate or other facial abnormalities

o   Will include “not for medication dispensing” in the use instructions and possibly on the cup itself

o   Will comply with all applicable US and European regulations for food contact materials

The proposed study will be conducted in Ghana, in its entirety. As the USFDA does not have jurisdiction in Ghana, the study is not subject to the USFDA regulations. We are consulting with the Ghana FDA to determine the documentation necessary to conduct this study in Ghana. The Ghana FDA has reviewed this protocol.

The generic cup that will be used in this study is a small medicine cup 30 ml in size (Figure 2).

Small medicine cups are manufactured by a variety of manufacturers and are commonly used in health facilities to feed breastmilk to infants who are having breastfeeding difficulties. The cups are generally translucent, calibrated with a variety of measurements including 2.5-30 mL.

Figure 2: Generic medicine cup
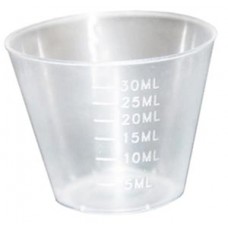


# 9.0 Risks

Potential study risks are minimal. We will not withhold any standard of care procedures. Our study procedures are non-invasive. Infants prescribed cup feeding by the clinical team will still be cup fed, which is the standard of care at KATH. The Nifty Feeding Cup has been evaluated in several infant populations in India and Tanzania and has been shown to be preferred by providers and caregiver over conventional cups (McKinney, et al, unpublished data). The Nifty Feeding Cup, with its unique design, minimizes typical problems with cup feeding such as spillage, coughing or aspirating on milk. Because generic cups pose the same risk and the NIFTY™ is specifically designed to reduce these risks, this research involves minimal risk.

Other risks pertain to the caregiver potentially feeling uncomfortable answering questions or being watched while feeding their infant. There is potential for lack of privacy around participation since we will conduct study visits at bedside. There is a risk of loss of confidentiality, though every effort will be made to protect patient identity.

# 10.0 Mitigation of risks

Caregivers will be provided a standardized set of instructions by the research assistant on how to cup feed prior to beginning to cup to feed their infant. This should minimize adverse effects of cup feeding in general. Caregivers/parents will be told that they are free to refuse to answer any item in any survey and free to decline participating in the study at any time and for any reason including lack of privacy without affecting their clinical care.

We will code all study data for each participant with a unique study identification number (ID). This will minimize use of personal identifiers but maintain the ability to link across sources of data collected from an individual study participant. We will use the Research Electronic Data Capture (REDCap) software to enter and store study data (see Data Capture Methods below). All electronic data will be protected using password-protected computers and will be protected by being stored on a HIPPA compliant server housed within the Institute for Translational Health Sciences (ITHS). Hard copy forms will be maintained in a locked filing cabinet. No individual-level data will be reported on study participants. These procedures will minimize the risk of loss of confidentiality.

# 11.0 Benefits

Caregiver-infant pairs may benefit by using the Nifty Feeding Cup and by being able to keep the Nifty Feeding Cup after the study for long-term use. Long term use of the Nifty Feeding Cup has the potential to improve health and reduce the risk of death. The additional benefits of this study to science, clinicians, participants, and the public are substantial. This will be the first comprehensive clinical study to examine the Nifty Feeding Cup. There are 9 million infants born with breastfeeding difficulties each year in South India and Africa. Most are preterm and preterm infants often have difficulty breastfeeding and need a supplement feeding tool. If effective, the Nifty Feeding Cup has widespread promise for being established as a standard of care cup for these infants with high impact to the health and survival of millions.

# 12.0 Study and safety monitoring

Safety monitoring for this study will focus on unanticipated problems (UP) involving risks to participants. Given the minimal risk nature of this study, unanticipated problems and serious adverse events (SAE) are highly unlikely. Nevertheless, oversight will be provided by the PIs, Drs. McKinney and Coffey and the site PI Gyikua Plange-Rhule. The Site PI will oversee the day-to-day activities of the study and report any potential adverse events to Drs. McKinney and Coffey who will be responsible for reporting.

The Study PIs (McKinney and Coffey) and the Site PI Gyikua Plange-Rhule will be jointly responsible for study oversight, including monitoring safety, ensuring that the study is conducted according to the protocol and ensuring data integrity. The PIs and study team will review the data for safety concerns and data quality at regular intervals and will promptly report to the IRB any UP that arises during the conduct of the study. A pediatrician at KATH will act as an independent safety monitor and will review any SAE (including research related injuries) that arise.

# 13.0 Managing and reporting adverse events

Serious Adverse Event (SAE). A serious adverse event (SAE) is a type of unanticipated problem one that in addition to meeting the UP definition, meets one or more of the following criteria:

- Results in death
- Is life-threatening (places the subject at immediate risk of death from the event as it occurred)
- Results in higher level inpatient hospitalization or prolongation of existing hospitalization
- Results in a persistent or significant disability or incapacity

An important medical event that may not result in death, be life threatening, or hospitalization may be considered an SAE when, based upon appropriate medical judgment, the event may jeopardize the subject and may require medical or surgical intervention to prevent one of the outcomes listed in this definition.

Incidents or events that meet the criteria for UPs or SAEs should include the following information when reporting to the IRB:

- appropriate identifying information for the research protocol, such as the title, investigator’s name, and the IRB project number;
- a detailed description of the adverse event, incident, experience, or outcome;
- an explanation of the basis for determining that the adverse event, incident, experience, or outcome represents a UP or SAE;
- a description of any changes to the protocol or other corrective actions that have been taken or are proposed in response.

# 14.0 Managing and reporting unanticipated problems or protocol deviations

Unanticipated Problems. Unanticipated problems involving risks to subjects or others to include, in general, any incident, experience, or outcome that meets all of the following criteria:

- unexpected in terms of nature, severity, or frequency given (a) the research procedures that are described in the protocol-related documents, such as the IRB-approved research protocol and informed consent document; and (b) the characteristics of the subject population being studied;
- related or possibly related to participation in the research (possibly related means there is a reasonable possibility that the incident, experience, or outcome may have been caused by the procedures involved in the research); and
- suggests that the research places subjects or others at a greater risk of harm (including physical, psychological, economic)

Incidents or events that meet the criteria for UPs or SAEs should include the following information when reporting to the IRB:

- appropriate identifying information for the research protocol, such as the title, investigator’s name, and the IRB project number;
- a detailed description of the adverse event, incident, experience, or outcome;
- an explanation of the basis for determining that the adverse event, incident, experience, or outcome represents a UP or SAE;
- a description of any changes to the protocol or other corrective actions that have been taken or are proposed in response.

# 15.0 Confidentiality and data management

15.1 Quality Control and Quality Assurance

### 15.1.1 Quality Assurance (QA)

QA activities such as study activities checklist, documentation of consent, immediate review of consent forms prior to initiating study procedures, and file reviews by research staff. Approximate bi-weekly meetings with the study team are planned. Site PIs will monitor the RA for compliance with standard hygiene practices in the mother-baby.

We will calibrate the scale weighing the milk using a scale at KATH to ensure measures are within 1 gram. We will do this on a monthly basis.

### 15.1.2 Quality Control (QC) and Auditing

QC will primarily focus on the accuracy of data entry into REDCap, the data management system, and establish methods for identifying inconsistent responses. Quality control of study procedures will be assured through the completion of training regimens specific to each study procedure. Audited CRFs will be double-entered and spot checked for consistency by the site PI or other study team member.

### 15.1.3 Staff Training

All research staff in this study will be trained in ethics and documentation of this training will be maintained as part of administrative study documents. Additional training identified by the site PI and required by KATH will be completed prior to initiating recruitment. Before recruiting or interacting with research subjects, staff will be trained by the PIs and/or clinical investigators on the protocol, manual of operations, and on study processes and procedures. New staff interactions with research subjects will be monitored. Follow-up training and monitoring will be on a case-by-case basis as needed. The RA will be trained in Infection Prevention and Control (IPC) and follow standard practices in the KATH mother-baby unit. The staff will be trained to wash his/her hands before measuring feeding cups, before touching the baby for anthropometry, and other contact with the mother-infant pair.

15.2 Data Handling and Record Keeping

### 15.2.1 Overview

The investigators and research staff are responsible for ensuring the accuracy, completeness, legibility, and timeliness of the data reported. All CRFs and source documents should be completed in a neat, legible manner to ensure accurate interpretation of data. The investigators will maintain adequate case histories of study participants, including accurate case report forms (CRFs).

### 15.2.2 Consent Documentation and Translation

The consent process will be documented in the research record. The consent form will be available in English and read to the caregiver as most will not be literate.

### 15.2.3 Data Corrections

Corrections to CRFs, consent forms and all other documents will be conducted by drawing a straight line through the incorrect information. Writing in the correct information and initialing and dating the correction.

### 15.2.4 Data Management Responsibilities

Data collection and accurate documentation are the responsibility of the study staff under the supervision of the investigators. All source documents must be reviewed by the study team and data entry staff, who will ensure that they are accurate and complete. UPs must be reported to an investigator who will review and take the needed action.

### 15.2.5 Data Capture Methods

We will capture all data electronically or on hard copy CRFs, based on the availability of computing resources needed (e.g. internet access). Data will be entered into REDCap either electronically or from hard copy forms. REDCap is a password-protected, web-based software tool for the collection and data entry of research data. Staff in Ghana will login to the REDCap project through a password protected REDCap portal hosted by the UW to enter data. The entered data is stored on a HIPAA compliant UW server. Data entry into REDCap of CRFs will be conducted on an ongoing basis. All records will be locked prior to final analysis.

### 15.2.6 Study Records Retention

Study records will be maintained for a minimum of five years from the date that the final report is submitted to Grand Challenges Canada. Study records will be deleted before or in 2025.

15.3 Participant Confidentiality and Data Storage

### 15.3.1 Participant Confidentiality

Participant confidentiality is strictly held in trust by the investigators and study staff. The study protocol, documentation, data, and all other information generated will be held in strict confidence. No data will be released to any unauthorized third party without prior written approval of the PIs.

### 15.3.2 Data Storage

Data will be shared by members of the research team. The PIs will control access to the data. Research data will be stored in a locked filing cabinet and/or in password protected computer files at KATH and data for each participant will be coded with a unique number. The key linking participant numbers to participant names will stored on a password-protected computer file with access limited to the study team. All electronic data will be stored on a password protected computer with access restricted to research team members. Data will be de-identified no later than **30 June 2025**. This timeframe was selected because studies and manuscripts generated often take longer than anticipated. De-identified data may be kept indefinitely.

# 16.0 Study costs

The participant will not incur any costs for participating in this study.

# 17.0 Care for injury

The study will pay for care from an injury that is research-related. Care for such injuries will be provided at KATH. The independent medical monitor will determine if a research-related injury has occurred by review records and discussing with the research staff.

# 18.0 Compensation

Caregiver who participate in the study will be given the Nifty Feeding Cup and the generic cup used in the study to keep and six diapers, as well as the bib cloth used to weigh spillage.

# 19.0 Investigator responsibilities

The PIs will be responsible for overseeing this study. Dr. McKinney will be in charge of training research staff on the study protocol and on how to conduct the study activities with study participants and will monitor data entry into REDCap. The site PI will be responsible for overseeing the research assistant conducting the study activities onsite and monitoring performance locally. Dr. Coffey will be primarily responsible for reporting to the funder.

# Appendices:

Provide separate attachments. Each attachment should have a footer/header with the document name, version number and date. Please attach:

1. Data collection forms, e.g., surveys, questionnaires, interview and focus group guides, eligibility checklists, adverse event report forms, case report forms.
   1. Baseline Survey
   2. Feeding Assessments (n=4)
   3. In-Hospital Preference Survey
   4. Chart Review of Discharge form
   5. Follow-up Survey (over the phone or in-person )
2. Consent form
   1. Consent (Caregiver)
   2. Photo Release Form (optional)
3. Recruitment materials.
   1. Recruitment Script
   2. Eligibility Checklist
   3. Screening and enrollment log
4. Study flow diagrams.
   1. Visit Activities Schedule
   2. Schematic of Study Procedures
5. Study products’ documentation (see section 8.0).

**Visit Activities Schedule**

| Procedure | Pre-Screening | Eligibility & Consent | V1. Baseline | V2. Cup 1 Feed 1 | V3. Cup 1 Feed 2 | V4. Cup 2 Feed 1 | V5. Cup 2 Feed 2 | Post Discharge | V6. Follow-up Survey |
| --- | --- | --- | --- | --- | --- | --- | --- | --- | --- |
| Recruitment |  |  |  |  |  |  |  |  |  |
| Prescreening log | X |  |  |  |  |  |  |  |  |
| Eligibility Checklist – Chart Review | X |  |  |  |  |  |  |  |  |
| Eligibility Checklist – Provider | X |  |  |  |  |  |  |  |  |
| Recruitment Script | X |  |  |  |  |  |  |  |  |
| Study Overview |  | X |  |  |  |  |  |  |  |
| Eligibility Checklist – Caregiver |  | X |  |  |  |  |  |  |  |
| Detailed review of consent |  | X |  |  |  |  |  |  |  |
| Obtain Signed Consent |  | X |  |  |  |  |  |  |  |
| Study Activities |  |  |  |  |  |  |  |  |  |
| Random Assignment |  |  | X |  |  |  |  |  |  |
| Baseline Survey |  |  | X |  |  |  |  |  |  |
| Cup 1 + Instructions |  |  | X |  |  |  |  |  |  |
| Measure infant weight/length |  |  | X |  |  |  |  |  |  |
| Feeding Assessment |  |  |  | X | X | X | X |  |  |
| Cup 2 + Instructions |  |  |  |  | X |  |  |  |  |
| Remove Cup 1 |  |  |  |  | X |  |  |  |  |
| In-Hospital Preference Survey |  |  |  |  |  |  | X |  |  |
| Chart Review of Discharge form |  |  |  |  |  |  |  | X |  |
| Follow-up Survey |  |  |  |  |  |  |  |  | X |

**Schematic of Study Procedures**

| Pre-Screening  Day -7 to 0 | - Identify potentials from (1) chart, (2) provider - Provider introduces study to caregiver/parent |
| --- | --- |
| Visit 1.  Eligibility and Consent /  Day 1, Hour 0 | - Study Overview - Screen Mother for Eligibility - Consent Process |
| Baseline  Day 1, Hour 0 | - Random assignment to Cup 1 - Baseline Survey - Infant weight, length - Provide Cup 1 + Instructions for Use |
| Visit 2.  Cup 1 Feed 1  >4 hours after V1 | - Complete Feeding Assessment |
| Visit 3.  Cup 1 Feed 2  >2 hours after V2 | - Complete Feeding Assessment - Remove Cup 1 - Provide Cup 2 + Instructions for Use |
| Visit 4.  Cup 2  Feed 1  >4 hours post V3 | - Complete Feeding Assessment |
| Visit 5.  Cup 2  Feed 2  [Final InfantVisit]  >2 hours post V4 | - Complete Feeding Assessment - Complete In-Hospital Preference Survey - Return Cup 1 to mother - Provide Thank You - Schedule Follow-up Survey |
| Post Discharge | - Abstract discharge date and health information (e.g. weight, feeding status ) onto Chart Review of Discharge form. |
| Visit 6.  Post-Discharge  4 weeks d/c | - Conduct Follow-up Survey 4 weeks post-discharge - Thank the participant verbally once more |

References

1. Lang S. Cup-feeding: An alternative method. *Midwives Chron Nurs Notes.* 1994;107:171-176.

2. World Health Organization. *Guidelines on optimal feeding of low-birthweight infants in low-and middle-income countries.* Geneva: WHO;2011.

3. WHO. *Managing newborn problems: a guide for doctors, nurses, and midwives.* Geneva, Switzerland: World Health Organization,;2003.

4. World Health Organization. *Breastfeeding Counselling A training course Trainer's Guide Part One.* WHO.

5. Glass RP, Wolf LS. Incoordination of sucking, swallowing, and breathing as an etiology for breastfeeding difficulty. *J Hum Lact.* 1994;10(3):185-189.

6. Kuehl J. Cup feeding the newborn: what you should know. *J Perinat Neonatal Nurs.* 1997;11(2):56-60.

7. Thorley V. Cup feeding: problems created by incorrect use. *J Hum Lact.* 1997;13(1):54-55.

8. Malhotra N, Vishwambaran L, Sundaram KR, Narayanan I. A controlled trial of alternative methods of oral feeding in neonates. *Early Hum Dev.* 1999;54(1):29-38.

9. Aloysius A, Hickson M. Evaluation of paladai cup feeding in breast-fed preterm infants compared with bottle feeding. *Early Hum Dev.* 2007;83(9):619-621.

10. Marinelli KA, Burke GS, Dodd VL. A comparison of the safety of cupfeedings and bottlefeedings in premature infants whose mothers intend to breastfeed. *J Perinatol.* 2001;21(6):350-355.

11. WHO. *Measuring a Child's Growth, Training Course on Child Growth Assessment: WHO Child Growth Standards, Sections 3 and 4.* Geneva, Switzerland: World Health Organization,;2008.

12. Schafer JL. Multiple imputation: a primer. Statistical Methods in Medical Research. Mar 1999;8(1):3-15.

13. Kathleen A. Leppig, Martha M. Werler, Cristina I. Cann, Catherine A. Cook, Lewis B. Holmes. Predictive value of minor anomalies. I. Association with major malformations. J Pediatr. 1987 Apr;110(4):531-7.

Modified Dubowitz assessment tool

| **Score** | **-1** | **0** | **1** | **2** | **3** | **4** | **5** |
| --- | --- | --- | --- | --- | --- | --- | --- |


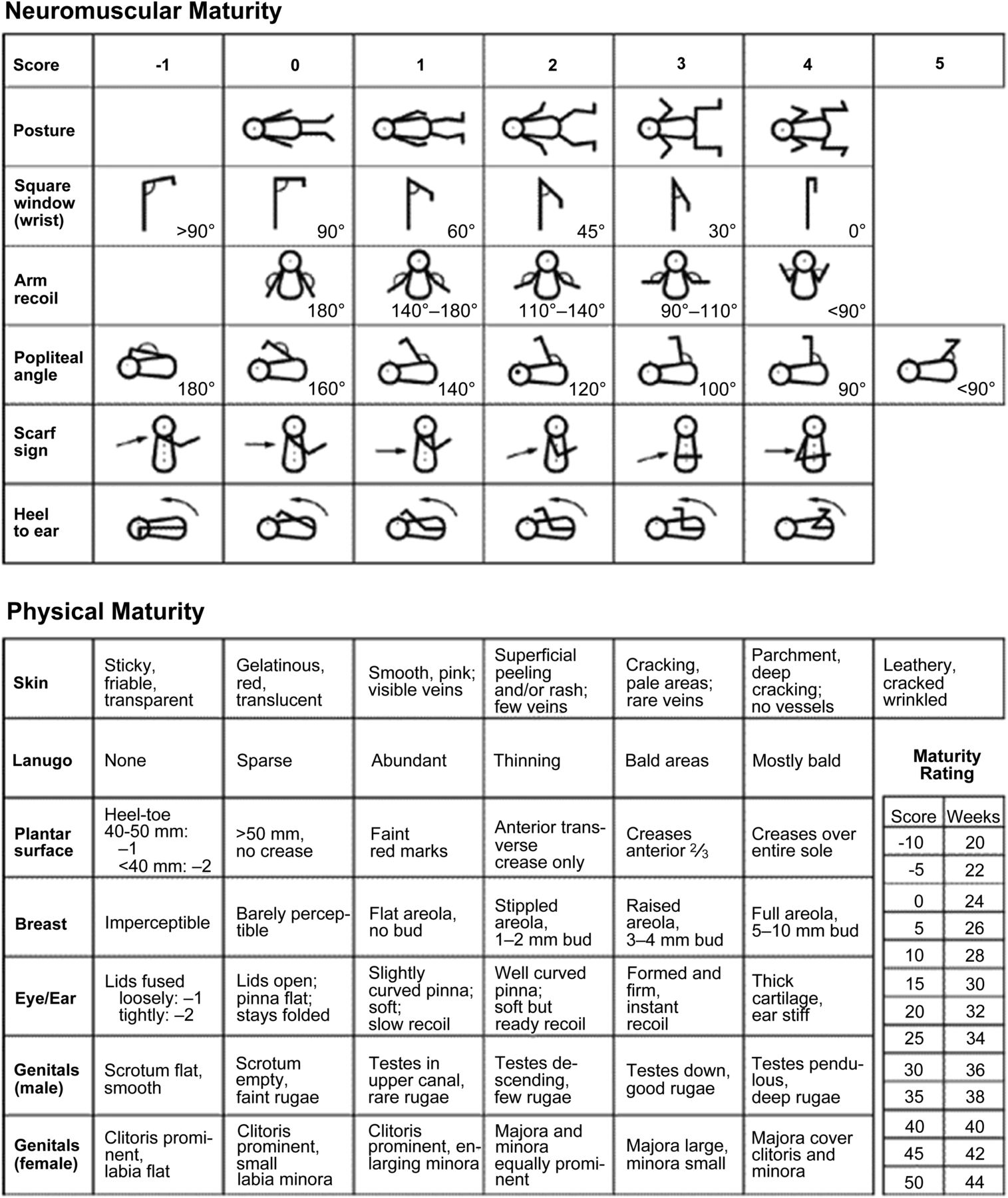

Supplement: S4 Appendix — (DOCX) [file pone.0223951.s007.docx]
